# PGFinder, a novel analysis pipeline for the consistent, reproducible, and high-resolution structural analysis of bacterial peptidoglycans

**DOI:** 10.7554/eLife.70597

**Published:** 2021-09-28

**Authors:** Ankur V Patel, Robert D Turner, Aline Rifflet, Adelina E Acosta-Martin, Andrew Nichols, Milena M Awad, Dena Lyras, Ivo Gomperts Boneca, Marshall Bern, Mark O Collins, Stéphane Mesnage

**Affiliations:** 1 School of Biosciences, University of Sheffield Sheffield United Kingdom; 2 Department of Computer Science, University of Sheffield Sheffield United Kingdom; 3 Institut Pasteur, Unité Biologie et Génétique de la Paroi Bactérienne Paris France; 4 INSERM, Équipe Avenir Paris France; 5 CNRS, UMR 2001 "Microbiologie intégrative et moléculaire" Paris France; 6 biOMICS Facility, Faculty of Science Mass Spectrometry Centre, University of Sheffield Sheffield United Kingdom; 7 Protein Metrics Inc Cupertino United States; 8 Infection and Immunity Program, Monash Biomedicine Discovery Institute Clayton Australia; 9 Department of Microbiology, Monash University Clayton Australia; Ecole Polytechnique Fédérale de Lausanne Switzerland; National Institute of Child Health and Human Development United States

**Keywords:** peptigoglycan, clostridium difficile, peptidoglycan structure, software, open source, jupyter notebook, *E. coli*, *C. difficile*

## Abstract

Many software solutions are available for proteomics and glycomics studies, but none are ideal for the structural analysis of peptidoglycan (PG), the essential and major component of bacterial cell envelopes. It icomprises glycan chains and peptide stems, both containing unusual amino acids and sugars. This has forced the field to rely on manual analysis approaches, which are time-consuming, labour-intensive, and prone to error. The lack of automated tools has hampered the ability to perform high-throughput analyses and prevented the adoption of a standard methodology. Here, we describe a novel tool called PGFinder for the analysis of PG structure and demonstrate that it represents a powerful tool to quantify PG fragments and discover novel structural features. Our analysis workflow, which relies on open-access tools, is a breakthrough towards a consistent and reproducible analysis of bacterial PGs. It represents a significant advance towards peptidoglycomics as a full-fledged discipline.

## Introduction

The characterisation of bacterial cell walls started with the development of electron microscopy techniques (Mudd and Lackman, 1941), and it has ever since been the focus of countless studies. The major and essential component of the bacterial cell envelope is called peptidoglycan (PG). It confers cell shape and resistance to osmotic stress and represents an unmatched target for antibiotics (Mainardi et al., 2008; Vollmer et al., 2008). Some of the most widely used antibiotics to date (beta-lactams and glycopeptides) inhibit the polymerisation of PG.

**Figure 1.**
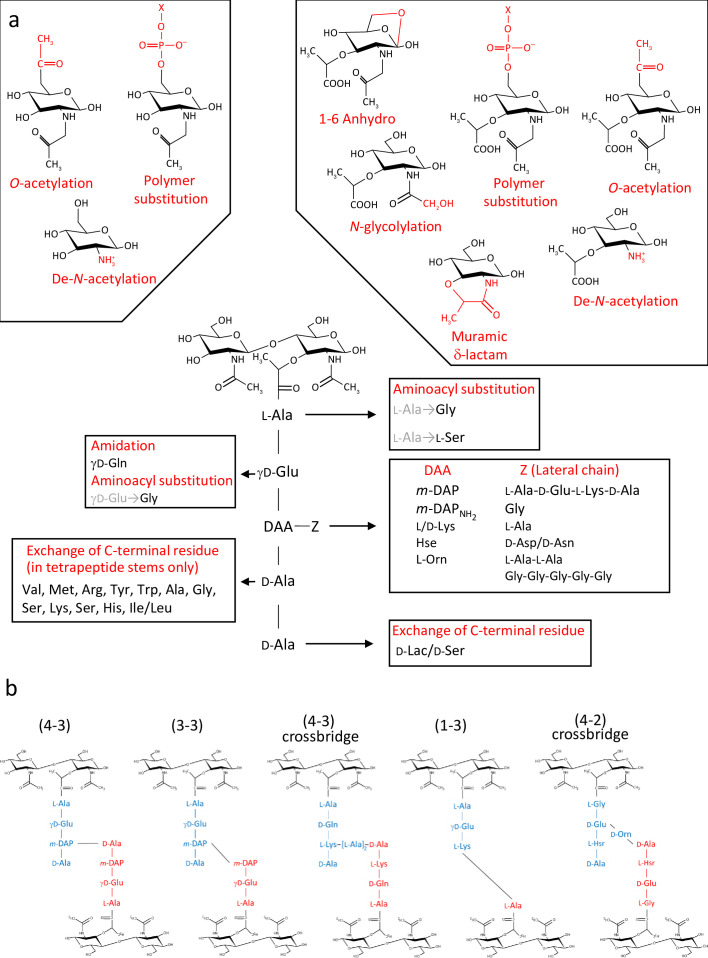
Diversity of peptidoglycan composition and structure. (**a**) Representative peptidoglycan building block made of *N*-acetylglucosamine (GlcNAc) and *N*-acetylmuramic acid (MurNAc) forming a disaccharide subunit linked to a pentapeptide stem attached to the MurNAc via a lactyl moiety. Peptide stem contains both L and D-amino acids and show a great diversity in composition. Some examples of amino acids found in peptidoglycan are shown for each residue. Modifications of the sugars are also shown. (**b**) Representation of crosslinking diversity, 4–3 bonds (direct or via peptide crossbridge) and 3–3 bonds are made by D,D- or L,D-transpeptidases, respectively. The enzymes catalysing 1–3 and 4–2 bonds remain unknown. Acceptors stems are shown in blue and donor stems in red. DAA: diamino acid; *m*-DAP: *meso*-diaminopimelic acid; D-Lac: D-lactate; X: cell surface polymer (e.g teichoic acid); Z: lateral chain.

PG (murein; originally known as mucopeptide) is a giant, insoluble, bag-shaped molecule, and its composition was characterised soon after its discovery (Cummins and Harris, 1956; Rogers and Perkins, 1959; Weidel and Pelzer, 1964). It is composed of glycan chains containing alternating *N*-acetylglucosamine (GlcNAc) and *N*-acetylmuramic acid (MurNAc) residues linked by β,1–4 bonds. The lactyl group of MurNAc residues is substituted by pentapeptide stems which often has the L-Ala_1_-γ-D-Glu_2_-L-DAA_3_-D-Ala_4_-D-Ala_5_ sequence, where DAA is a diamino acid such as *meso*-diaminopimelic (mDAP) acid or L-lysine (Figure 1a; Vollmer et al., 2008). In some species, a lateral chain (with variable composition and length) can be found attached to the amino acid in position 3. Peptide stem composition and polymerisation can vary amongst bacterial species (Schleifer and Kandler, 1972). Whilst PG building blocks produced in the cytoplasm are always the same, the final structure undergoes constant lysis and modification, a process referred to as ‘remodelling’. Both remodelling and alternative polymerisation modes (Figure 1b) lead to a considerable variation in PG structure during cell growth and division. PG structural plasticity plays a critical role for adaption to environmental conditions during host-pathogen interaction (Boneca et al., 2007; Juan et al., 2018) or to survive exposure to antibiotics (Mainardi et al., 2008).

PG material is straightforward to purify, but the structural analysis of this molecule is challenging and remains a time-consuming and labour-intensive process. The intact molecule must be broken down into soluble fragments by enzymatic digestion with a glycosyl hydrolase (lysozyme), and individual building blocks (disaccharide peptides, also called muropeptides) are analysed to gain insight into the structure of the intact molecule. A transformative step for the characterisation of disaccharide peptides has been the use of reversed-phase HPLC (rp-HPLC) and mass spectrometry (MS) towards the end of the 1990s (Garcia-Bustos et al., 1988; Glauner, 1988; Glauner et al., 1988; Martin et al., 1987). Combining muropeptides separation by rp-HPLC and MS characterisation has hinted at a more complex structure than previously reported.

Despite tremendous advances in both rp-HPLC-MS instrumentation and software development for the automated analysis of large datasets, ‘peptidoglycomics’ is still in its infancy. The experimental strategy to analyse PG structure has barely changed over the past 30 years. Even though rp-HPLC-MS has been routinely used over the past decade, the analysis of MS data remains a black box. Except for a recent study describing the PG structure of *Pseudomonas aeruginosa* (Anderson et al., 2020b), no information is available in the literature about the strategy used to identify muropeptides in rp-HPLC-MS datasets. This task relies on searching a subset of expected structures, but the complexity of both the search space and search process is often not described.

We previously provided the proof of concept that shotgun proteomics tools can be used for the automated and unbiased analysis of PG structure (Bern et al., 2017). The analysis of *Clostridioides* (previously *Clostridium*) *difficile* PG led to the identification of many muropeptides never reported before. This work also demonstrated that PG analysis could be carried out with relatively high throughput, opening the possibility to analyse large numbers of samples (such as clinical or environmental isolates) using a minimal amount of material (typically microgram amounts).

Here, we describe a novel software called PGFinder for the analysis of MS data. PGFinder is a versatile and straightforward open-source software tool that allows automated identification of muropeptides based on the creation of dynamic databases. Sharing PGFinder as a Jupyter Notebook provides a robust and consistent pipeline with the potential to accelerate discovery in the field of peptidoglycomics. This workflow described here allows a comprehensive description of the analysis strategy for a consistent and reproducible PG structure analysis by users in the community.

We applied the PGFinder pipeline to analyse the muropeptides composition of *Escherichia coli* which has been extensively studied. We demonstrate that PGFinder can capture an unprecedented level of complexity of PG structure, highlighting the limitations of the search strategies reported so far. Finally, we provide evidence that PGFinder can be used in conjunction with freely available MS data deconvolution software, making PG analysis possible using entirely open-access tools. We propose that our approach represents a significant advance towards a consistent and reproducible analysis of PG structure, allowing peptidoglycomics to take the crucial first leap to parity with other omics disciplines.

## Results

### PGFinder: a dedicated script for bottom-up identification of PG fragments

No pipeline is currently available for the automated analysis of MS PG data. Therefore, we sought to replicate a shotgun proteomics approach to create an analysis pipeline dedicated to PG analysis, referred to as ‘peptidoglycomics’ (Wheeler et al., 2014).

**Figure 2.**
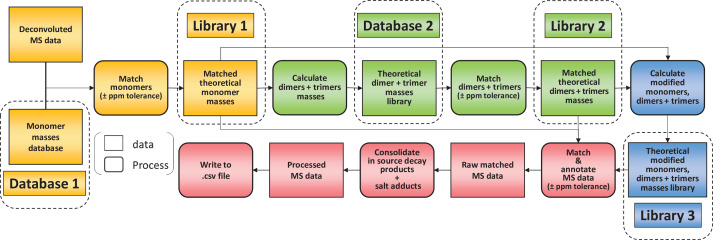
Flowchart outlining the algorithm for the matching script. The identification of muropeptides was carried out using four successive steps, indicated by different colours (orange, green, blue, and red, respectively). As a first step, observed masses in the dataset are compared to a list of theoretical masses corresponding to monomers (database 1). Matched masses within the ppm tolerance set (10 ppm for Orbitrap data) are used to build a list of inferred monomeric structures and their corresponding theoretical masses (library 1). This is then used to generate a list of theoretical multimers (dimers and trimers) and their masses (database 2). A second matching round is carried out to build a list of inferred multimers (library 2). At this stage, matched monomers and multimers are combined to generate a list of modified muropeptides (library 3). Two libraries of matched theoretical masses (monomers and dimers, trimers) and a third library (their modified counterparts) are used to search the dataset. Muropeptide structures are inferred from a match within tolerance between theoretical and observed masses. This data is then ‘cleaned up’ by combining the intensities of ions corresponding to in-source decay and salt adducts to those of parent ions. The final matched mass spectrometry data is then written to a .csv file.

To limit misidentifications due to mass coincidences, we established a search strategy relying on an iterative process (Figure 2). A first search was carried out using a database made of reduced disaccharide peptides (monomers) and their theoretical monoisotopic masses. MS data were deconvoluted using the Protein Metrics Byos software to generate a list of observed monoisotopic masses alongside other parameters including retention times and signal intensity. Individual theoretical masses contained in the monomer database (Figure 2, database 1) were compared with observed masses in the experimental dataset. Any observed mass within 10 ppm tolerance was considered as a match and the corresponding inferred structure and theoretical mass were then added to a list of matched structures (Figure 2, library 1). As a second step, we used the list of matched monomers to build another database in silico (Figure 2, database 2), corresponding to dimers and trimers and their theoretical masses. Two types of polymerisation events are included in the original PGFinder version depending on the type of crosslink either through peptide stems or glycan chains. Individual theoretical masses from the in silico database were compared to observed masses to generate a list of matched dimers and trimers (Figure 2, library 2). As a third step, we combined the lists of matched monomers and multimers to generate a final library of modified muropeptides (Figure 2, library 3). The final library contained only modified muropeptides corresponding to matched monomers, dimers, and trimers. The modifications accounted for include the presence of anhydro groups, deacetylated sugars, amidated amino acids, and modifications resulting from *N*-acetylglucosaminidase or amidase activities (loss of GlcNAc and lack of peptide stems, respectively). In-source decay products (loss of GlcNAc) and Na^+^/K^+^ salt adducts were also added to library 3. All three libraries corresponding to observed monomers, dimers, trimers, and their modified variants were combined to search the MS data for masses matching theoretical values within a 10 ppm mass accuracy window. This search generated results processed by PGFinder to carry out a ‘clean up step’. The intensities of in-source decay products and salt adducts were combined with that from parent ions when found within close retention time (a 0.5 min time window). The output of this final step is a matched table written to a .csv format file. It contained all the inferred structures identified within the specified mass and retention time windows with an extracted-ion chromatogram (XIC) signal intensity for quantification.

### Using PGFinder to investigate PG structure and identify low-abundance muropeptides

**Figure 3.**
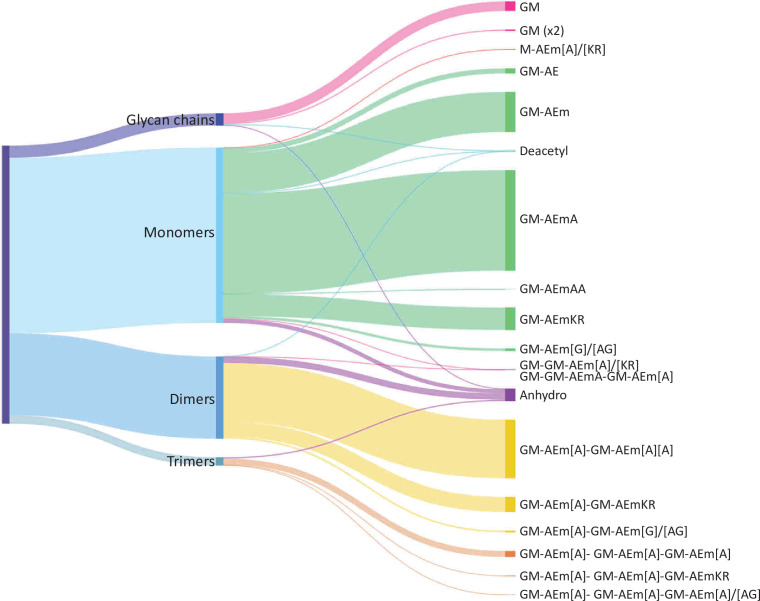
Distribution of *E. coli* peptidoglycan fragments identified using automated search workflow. Breakdown of peptidoglycan is shown by oligomerisation state (left) branching to specific composition (right). Branch size is proportional to percentage. Monomers, dimers, trimers, and glycan chains (left) are broken down into muropeptide composition and structure (right). Individual structures are grouped by colour according to oligomerisation state. Monomers, green; dimers, yellow; trimers, orange. Residues in square brackets are only found in some muropeptides. For example, GM-AEJ[A]-GM-AEJ[A] can represent GM-AEJA-GM-AEJA, GM-AEJA-GM-AEJ, and GM-AEJ-GM-AEJ. G: *N*-acetylglucosamine; M, *N-*acetylmuramic acid; A: L- or D- alanine; E: γ-D-glutamic acid; J: *meso*-diaminopimelic acid; K: D-lysine; R: D-arginine; G: glycine.

The performance of the matching script was tested using the well-characterised PG from *E. coli* as a proof of concept. UHPLC-MS/MS data were acquired for three independent PG samples (biological replicates; Appendix 1—figure 1). Following MS1 spectral deconvolution (a process calculating masses from observed *m/z* values), observed masses were matched to theoretical muropeptides masses according to the strategy described above (Figure 2). A first search was carried out using a minimal mass database made of 10 simple PG fragments including three glycan chains (di-, tetra-, and hexasaccharides) and seven monomers (Table 1—source data 1). Due to the .csv format of the database, the diamino acid in position 3 could not be assigned to a symbol or Greek letter and had to be one of the 26 letters already assigned by the IUPAC-IUB Joint Commission on Biochemical Nomenclature. We used the letter J for mDAP for the initial search and replaced it by the letter m in the final table. The output of the automated search is a .csv file per dataset; all files corresponding to biological replicates were collated into one Excel file (Table 1—source data 2). Each search output contained approximately 3000 rows of masses and corresponding parameters. Depending on the dataset analysed, 41–48% of the total ion intensity was assigned to PG structures. As anticipated, inferred structures were frequently found with multiple retention times, reflecting the existence of stereoisomers, with one species accounting for most of the intensity. In some cases, observed masses matched with more than one inferred structure. The output of the automated search was consolidated as described in Supplementary file 1. Retention times were assigned to individual structures based on the elution of the most abundant stereoisomer. For example, >97% of the most abundant monomer GM-AEJA was eluted at an average retention time of 10.04 ± 0.04 min. Data consolidation revealed an unprecedented muropeptide composition complexity compared to recent LC-MS analyses of *E. coli* (Kühner et al., 2014; Table 1—source data 2). Sixty PG fragments were identified (Table 1): these included glycan chains lacking peptide stems (4.38%), monomers (63.14%), dimers (29.54%), and trimers (2.94%) (Figure 3). Based on the abundance of multimeric PG fragments, we report a crosslinking index of 15.69%, which is slightly lower than the value previously reported of 23.1% (Glauner, 1988).

**Table 1.** Processed match output. Table 1—source data 1.*E. coli* simple mass database.
Table 1—source data 2.*E. coli* matching output and consolidated data.
Table 1—source data 3.MS/MS analysis of *E. coli* glycan chains and monomers.
Table 1—source data 4.*E. coli* complex mass database.
Table 1—source data 5.*E. coli* muropeptide complex table.

	Structure	RT (min)	Abundance (%)	Monoisotopic mass (Da)		
		Av±SD	Av±SD	Obs	Theo	Δppm
	GM|0	3.62±0.01	3.465±0.683	498.205	498.206	2.5
Glycans	GM (x2)|0	10.11±0.03	0.428±0.349	976.384	976.386	2.2
4.38%±0.35%	GM (anhydro) |0	8.20±1.92	0.238±0.025	478.179	478.180	2.9
	GM (deacetyl) |0	2.57±0.00	0.155±0.032	456.194	456.196	3.5
	GM (x2) (deacetyl) |0	6.86±0.02	0.093±0.012	934.372	934.376	3.2
	GM-AEmA|1	10.04±0.04	36.098±2.131	941.405	941.408	2.8
	GM-AEm|1	6.57±0.01	14.352±0.397	870.368	870.371	3.0
	GM-AEmKR|1	9.56±0.05	8.030±0.774	1154.563	1154.567	3.6
	GM-AE|1	9.57±0.04	1.809±0.231	698.284	698.286	3.1
	GM-AEmG|1	7.85±0.05	0.689±0.049	927.390	927.392	2.3
	GM-AEm (anhydro) |1	13.98±0.02	0.668±0.073	850.342	850.344	2.2
Monomers	GM-AEmA (anhydro) |1	16.55±0.01	0.573±0.100	921.380	921.382	2.0
63.14%±1.13%	GM-AEmAG|1	9.45±0.05	0.219±0.009	998.426	998.429	3.1
	GM-AEmKR (anhydro) |1	14.83±0.01	0.160±0.039	1134.537	1134.540	2.9
	GM-AEmA (deacetyl) |1	8.57±0.06	0.083±0.055	899.394	899.397	3.1
	GM-GM-AEmA|1	13.10±0.02	0.075±0.040	1419.584	1419.588	2.9
	GM-AE (anhydro) |1	17.44±0.01	0.069±0.013	678.258	678.260	2.8
	M-AEm|1	4.56±0.01	0.062±0.064	667.289	667.291	3.8
	M-AEmKR|1	8.16±0.06	0.061±0.056*	951.484	951.487	3.2
	GM-AEmAA|1	11.38±0.04	0.059±0.003	1012.442	1012.445	2.4
	M-AEmA|1	8.52±0.05	0.053±0.015	738.325	738.328	4.0
	GM-GM-AEm|1	11.31±0.04	0.042±0.025	1348.547	1348.551	2.4
	GM-AEm (deacetyl) |1	4.77±0.01	0.024±0.014	828.358	828.360	3.0
	GM-GM-AEmKR|1	12.18±0.03	0.011±0.002*	1632.742	1632.747	3.0
	GM-AEmA-GM-AEmA|2	16.01±0.02	17.247±0.777	1864.800	1864.805	2.3
	GM-AEmA-GM-AEmKR|2	14.83±0.02	4.589±0.589	2077.957	2077.964	3.0
	GM-AEmA-GM-AEm|2	15.09±0.02	3.207±0.168	1793.763	1793.768	2.6
	GM-AEmA-GM-AEmA (anhydro) |2	20.56±0.01	0.873±0.037	1844.774	1844.778	2.4
	GM-AEm-GM-AEmKR|2	14.22±0.00	0.855±0.101	2006.920	2006.926	3.3
	GM-AEmA-GM-AEmKR (anhydro) |2	18.89±0.17	0.665±0.079	2057.934	2057.937	1.8
	GM-AEm-GM-AEm|2	14.23±0.01	0.558±0.062	1722.725	1722.730	3.0
	GM-AEm-GM-AEmAG|2	14.68±0.01	0.416±0.025	1850.785	1850.789	2.4
	GM-AEmA-GM-AEm (anhydro) |2	19.66±0.01	0.381±0.028	1773.738	1773.741	2.1
Dimers	GM-AEmA-GM-AEmAG|2	15.33±0.02	0.179±0.005	1921.822	1921.826	2.2
29.54%±0.46%	GM-AEm-GM-AEmKR (anhydro) |2	18.07±0.01	0.170±0.024	1986.896	1986.900	2.1
	GM-AEm-GM-AEm (anhydro) |2	18.77±0.01	0.141±0.015	1702.697	1702.704	4.5
	GM-AEmA-GM-AEmAA|2	16.54±0.01	0.075±0.002	1935.838	1935.842	2.1
	GM-AEm-GM-AEmG|2	13.91±0.01	0.054±0.003	1779.747	1779.752	2.7
	GM-GM-AEmA-GM-AEmA|2	17.51±0.01	0.046±0.028	2342.976	2342.985	3.6
	GM-AEmA-GM-AEmA (deacetyl) |2	15.17±0.01	0.029±0.022	1822.789	1822.794	3.0
	GM-AEmA-GM-AEmG (anhydro) |2	19.12±0.01	0.021±0.001	1830.761	1830.763	0.7
	GM-AEmA-GM-AEmAG (anhydro) |2	19.73±0.01	0.019±0.002	1901.796	1901.800	2.1
	GM-AEmA-GM-AEmAA (anhydro) |2	21.17±0.02	0.015±0.002	1915.812	1915.816	1.8
	GM-GM-AEmA-GM-AEm|2	16.85±0.00	0.003±0.004	2271.943	2271.947	2.1
	GM-AEmA-GM-AEmA-GM-AEmA|3	18.86±0.01	1.751±0.221	2788.192	2788.202	3.5
	GM-AEmA-GM-AEmA-GM-AEm|3	18.23±0.21	0.371±0.031	2717.158	2717.164	2.2
	GM-AEmA-GM-AEmA-GM-AEmA (anhydro) |3	22.39±0.02	0.222±0.027	2768.169	2768.175	2.3
	GM-AEmA-GM-AEmA-GM-AEmKR|3	17.54±0.01	0.207±0.028	3001.350	3001.360	3.4
	GM-AEmA-GM-AEmA-GM-AEm (anhydro) |3	21.60±0.02	0.117±0.003	2697.133	2697.138	1.8
Trimers	GM-AEmA-GM-AEmA-GM-AEmKR (anhydro) |3	20.90±0.16	0.088±0.026	2981.328	2981.334	2.2
2.94%±0.36%	GM-AEmA-GM-AEmA-GM-AEmG|3	17.72±0.01	0.039±0.004	2774.182	2774.186	1.4
	GM-AEmA-GM-AEm-GM-AEm|3	17.45±0.01	0.029±0.005	2646.123	2646.127	1.7
	GM-AEmA-GM-AEm-GM-AEm (anhydro) |3	21.16±0.01	0.025±0.001	2626.096	2626.101	1.9
	GM-AEmA-GM-AEm-GM-AEmKR|3	17.11±0.01	0.022±0.002	2930.316	2930.323	2.7
	GM-AEmA-GM-AEmA-GM-AEmAG|3	18.24±0.01	0.021±0.001	2845.217	2845.223	2.0
	GM-AEmA-GM-AEmA-GM-AEmAA|3	19.23±0.01	0.014±0.002*	2859.235	2859.239	1.3
	GM-AEmA-GM-AEm-GM-AEmKR (anhydro) |3	20.31±0.02	0.014±0.005	2910.293	2910.297	1.5
	GM-AEm-GM-AEmG-GM-AEmAG|3	17.18±0.00	0.004±0.005	2703.143	2703.149	2.0
	GM-AEmA-GM-AEm-GM-AEmG (anhydro) |3	21.21±0.02	0.011±0.003*	2754.157	2754.160	1.1
	GM-AEmA-GM-AEmA-GM-AEmAG (anhydro) |3	21.77±0.01	0.006±0.004	2825.189	2825.197	2.8

Inferrred dimers and trimers are based on the most abundant monomers and could correspond to alternative structures.

G: GlcNAc; M: MurNAc; m: *meso*-diaminopimelic acid; the number following the symbol ‘|’ refers to the oligomerisation state (1 for monomers, 2 for dimers, and 3 for trimers).

* Calculated from two values.

The automated and unbiased search revealed several muropeptides that were expected but never reported to date for *E. coli*. These included (i) PG fragments resulting from amidase activity (4.55%), found as ‘denuded glycans’ (disaccharides and tetrasaccharides) and modified variants or muropeptide stem with an extra disaccharide residue; (ii) a low-abundance (0.23%) PG fragments containing deacetylated GlcNAc residues; and (iii) PG fragments resulting from glucosaminidase activity (0.12%). Deacetylated muropeptides were not expected since no *E. coli* PG deacetylase has been identified in this organism to date. All the structures identified for the first time (glycan chains, monomers containing deacetyl groups, and muropeptides lacking a GlcNAc residue) were confirmed by MS/MS analysis (Table 1—source data 3). The proportion of muropeptides with anhydroMurNAc groups identified (4.55%) was in line with previous studies, yielding an average chain length of 36.05. This value is higher than an earlier study that reported a predominant chain length of 5–10 disaccharide units (Harz et al., 1990), but agreed with recent work that reported long glycan chains in *E. coli* (Turner et al., 2018). Overall, the quantification of muropeptides across biological replicates was very consistent, with Pearson’s correlation coefficients >0.96 (Appendix 1—figure 2). The most pronounced variations in quantification were observed with low-abundance muropeptides accounting for less than 1% of the species identified.

We further explored the structural diversity of *E. coli* PG, performing a more complex search with a mass database made of glycan chains and all possible monomers containing di-, tri-, tetra-, and pentapeptide stems (Table 1—source data 4; 224 structures in total). Several monomers with tetra- and pentapeptide stems containing unusual amino acids were identified. Only four structures could be confirmed by MS/MS analysis and corresponding multimers were retained (GM-AEJF, -AEJN, AEJK, -AEJAK, and GM-AEJKD). Collectively, muropeptides containing unusual amino acids accounted for ca. 7.5% of the 80 structures identified (Table 1—source data 5) using a complex mass database.

### Using PGFinder for the comparative analysis of PG structures

We showed with *E. coli* data that PGFinder is suitable to characterise the high-resolution structure of PGs using a ‘bottom-up’ approach. However, this requires a careful analysis of the search output to confirm the identity of muropeptides identified and discriminate between multiple structures that can be assigned to a unique observed mass. A more basic application is the use of PGFinder in organisms that have already been studied in detail to either compare PG composition or quantify the abundance of specific structures. This application accounts for most PG analyses described in the literature, comparing PG structures between different isolates, isogenic mutants, or cells grown in different conditions.

We chose the PG of *C. difficile* as a proof of concept to demonstrate how PGFinder can carry out a straightforward comparative analysis. We prepared PG samples corresponding to biological triplicates from two clinical isolates, R20291 and M7404. To illustrate the versatility of the software, PG samples were digested by mutanolysin, and disaccharide peptides were beta-eliminated to generate lactyl-peptides (Tipper, 2002) and analysed by UHPLC-MS (Figure 4—figure supplement 1). Since the high-resolution PG structure of *C. difficile* has been described based on the MS/MS analysis of muropeptides (Bern et al., 2017), we used data previously published to build a PG fragment database for a ‘one-off’ matching step. The database included monomers identified by MS/MS, containing unusual amino acids and the corresponding dimers resulting from either D,D- or L,D-transpeptidation (with a AEJA or AEJ peptide as donor stem). To limit the complexity of the search output, we limited the list of trimers, tetramers, and pentamers to those containing the most abundant peptide stems found in dimers (AEJA, AEJ, or AEJG). The database of theoretical masses contained 74 PG structures described in Figure 4—source data 1. MS data were deconvoluted using Byos Feature Finder, and observed monoisotopic masses were matched to theoretical masses using PGFinder. To perform the matching operation in its simplest form, all options offered by the software were deactivated.

**Figure 4.**
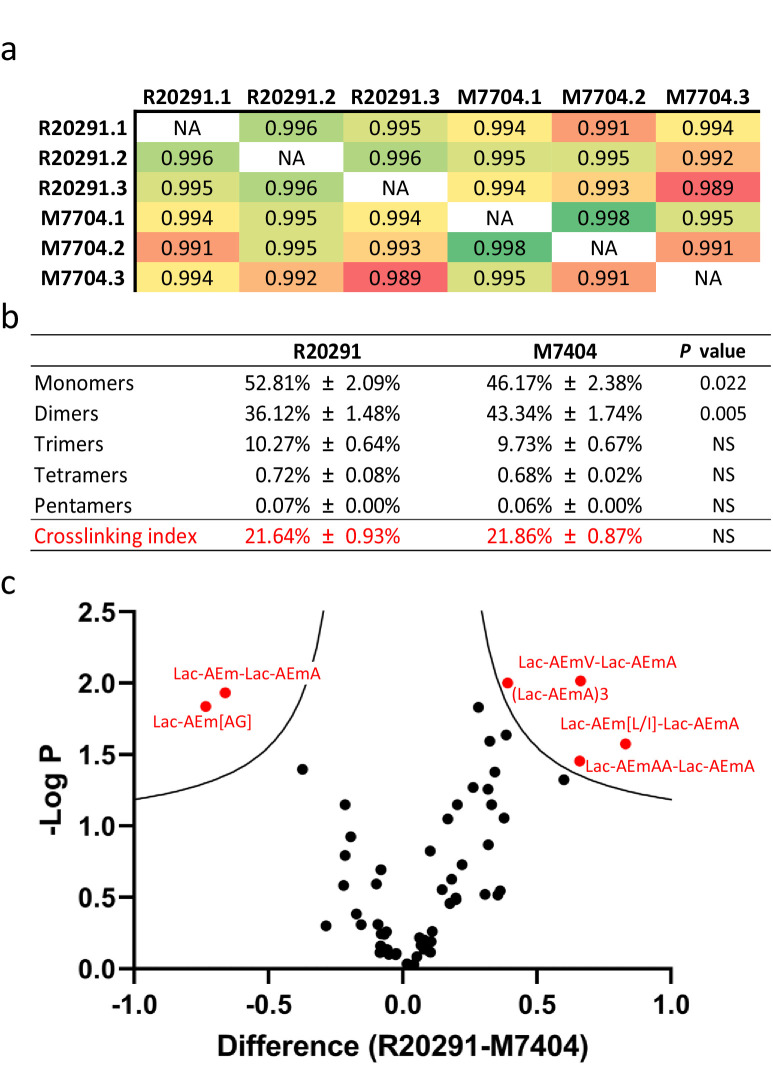
Comparative analysis of *C. difficile* R20291 and M7404 peptidoglycan (PG) composition. (**a**) Pearson’s correlation coefficients across biological replicates of R20291 and M7404 *C. difficile* isolates. Heatmap gradient shows highest value in green to lowest value in red. (**b**) Muropeptide distribution according to degree of crosslinking. Comparison was carried out using a Student’s *t*-test; p-value is indicated for each category of muropeptides. (**c**) Volcano plot, where each dot represents an individual muropeptide, plotted against the significance (Student’s *t*-test p-value<0.05, FDR < 0.05, S_0_ = 0.1) and difference (log_2_). Muropeptides showing a significantly different abundance between strains are highlighted in red. Lac: lactyl group; A: D/L-alanine; E: γ-D-glutamate; J: *meso*-diaminopimelic acid V: D-valine; L: D-leucine; I: D-isoleucine; G: glycine. Figure 4—source data 1.*C.* *difficile* mass database. Figure 4—source data 2.*C.* *difficile* 20291 versus M7404, list of muropeptides, abundance, RT.

All monomers and dimers searched were identified, except for two muropeptides containing a peptide stem with a methionine residue in position four, both present in low abundance in *C. difficile* strain 630 (ca. 0.10%). 25 out of the 31 possible trimers, tetramers, and pentamers searched were found (Figure 4—source data 2). Comparison of biological replicates revealed a high reproducibility, both in retention times and quantification. A high correlation was found between biological replicates, confirming the robustness of the quantification method (Figure 4a). Both strains contained a similar amount of mono-, tri-, tetra-, and pentamers, but strain M7404 contained a significantly lower proportion of monomers and a higher proportion of dimers than strain R20291 (p=0.022 and p=0.005, respectively; Student’s *t*-test; Figure 4b). We next performed a Student’s *t*-test using permutation-based FDR to identify statistically significant differences in the abundance of individual muropeptides between the two strains. The p-value was plotted on a volcano plot against the fold change in abundance between the two samples (Figure 4c). Two muropeptides were significantly less abundant (Lac-AEJ[AG] and Lac-AEJ-Lac-AEJA), and four others were significantly more abundant in strain R20291 (Lac-AEJV- Lac-AEJA, Lac-AEJ[L/I]-Lac-AEJA, Lac-AEJAA-Lac-AEJA and the trimer (Lac-AEJA)3). These differences are likely to reflect different substrate specificities for PBPs and the Ddl ligases in these strains. Therefore, combining the output of PGFinder with statistical analysis of muropeptide abundance offers a robust workflow to identify differences in PG composition.

**Figure 4—figure supplement 1.**
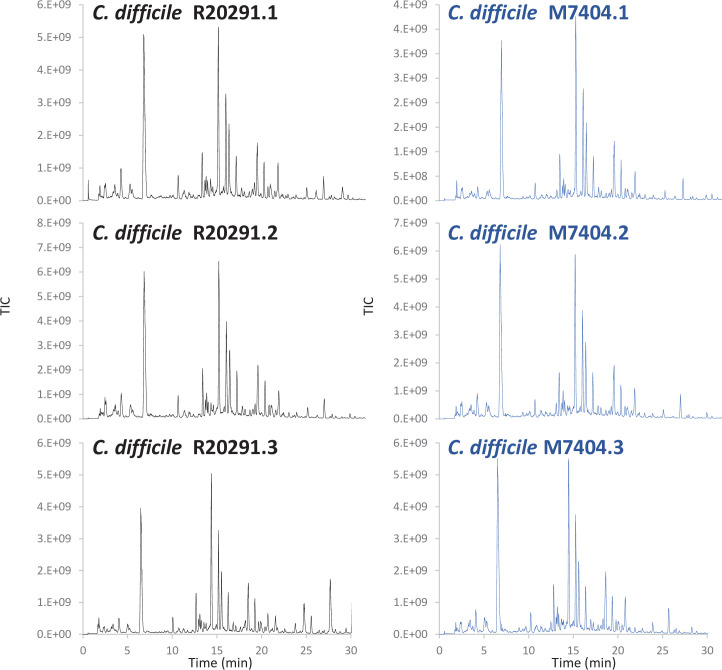
*C.* *difficile* LC-MS chromatograms.

### Benchmarking the automated PG analysis pipeline using available datasets

The analysis of *E. coli* PG established a proof of concept, showing that our matching script is suitable for the automated analysis of PG MS data. We next sought to evaluate the robustness of our peptidoglycomics pipeline using available datasets described in the literature. The most suitable publication was a recent study by Anderson et al. describing a PG analysis of *P. aeruginosa* planktonic cells (Anderson et al., 2020b). Unlike most (if not all) studies published to date, this work provided datasets from biological and technical triplicates. Unlike our *E. coli* samples, analysed on a Q Exactive Focus Orbitrap (Thermo), *P. aeruginosa* samples were analysed on an Agilent Q-TOF mass spectrometer. Spectra were deconvoluted using the Byos Feature Finder module, and observed masses were matched using a mass tolerance of 25 ppm as described by Anderson et al. To limit the occurrence of mass coincidences and misidentification at this slightly lower mass accuracy, we carried out a search with PG modifications (anhydroMurNAc residues, deacetylation, lack of peptide stem resulting from amidase activity and amidation) but only using the most frequent combinations of modifications (double anhydroMurNAc and anhydroMurNAc and deacetyl).

PGFinder identified 63 muropeptides out of the 71 reported by Anderson et al., matching our search criteria (Table 2). The eight muropeptides that were not identified were absent from the list of deconvoluted masses, indicating that the problem was not associated with the script, highlighting that the deconvolution step is a source of variability. Interestingly, the observed masses calculated using Byos Feature Finder were closer to the theoretical value (6.5 ppm versus 10.7 ppm on average), reflecting another source of variability associated with data deconvolution. Four muropeptides previously identified containing the AEJAG pentapeptide stem were matched with distinct structures due to a mass coincidence between K and AG. A careful analysis of MS/MS spectra suggested that these muropeptides contained a K residue rather than the AG dipeptide (the y2 ion being 143 ppm away from the expected mass), showing the added value of an unbiased search. This conclusion is supported by the retention times of the corresponding muropeptides since the tetrapeptide AEJK elutes before EAJA whilst the pentapeptide AEJAG elutes later in the chromatography (Bern et al., 2017). It is worth noting that our search also identified a large number of muropeptides that were not reported previously (Table 2—source data 1). These results collectively show that our PG analysis pipeline can identify an unprecedentedly large number of muropeptide structures based on MS1 data, including all those previously reported (Anderson et al., 2020a; Anderson et al., 2020b).

**Table 2.** Automated identification of *P. aeruginosa* peptidoglycan fragments. Table 2—source data 1.*Pseudomonas aeruginosa* matched muropeptides not reported previously.
Table 2—source data 2.Raw output of automated search using MaxQuant and PGFinder.

Inferred structure	Mass		∆ppm		MaxQuant
	Theoretical	Observed	This work	Anderson et al.	
GM (anhydro)	478.1799	478.1780	4.0	–2.7	+
GM	498.2061	498.2042	3.9	–4.2	+
GM (x2) (deacetyl)	934.3755	934.3706	5.3	–8.6	+
GM (x2) (anhydro)	956.3598	956.3551	5.0	6.0	+
GM (x2)	976.3860	976.3794	6.7	–6.1	+
GM (x3) (deacetyl)	1412.5554	1412.5490	4.5	–6.2	+
GM (x3) (anhydro)	1434.5397	1434.5348	3.4	–7.5	+
GM (x3)	1454.5659	1454.5592	4.6	–5.3	+
GM (x4)	1932.7458	1932.7352	5.5	–5.1	+
GM-AE (anhydro)	678.2596	678.2567	4.3	–9.1	+
GM-AE	698.2858	698.2830	3.9	–12.9	+
GM-AEJ (anhydro)	850.3444	850.3401	5.1	–10.6	+
GM-AEJ	870.3706	870.3676	3.5	–5.9	+
GM-AEJA (anhydro)	921.3815	921.3765	5.4	–9.9	+
GM-AEJG	927.3920	927.3868	5.6	–8.9	+
GM-AEJA	941.4077	941.4045	3.4	–5.0	+
GM-AEJC	973.3843	973.3763	8.2	–2072.2	+
GM-AEJL	983.4593	983.4498	9.6	–15.5	+
GM-AEJK	998.4703	998.4624	8.0	–10.6	+
GM-AEJM	1001.4153	1001.4060	9.2	–13.5	+
GM-AEJAA	1012.4448	1012.4413	3.4	–7.8	+
GM-AEJY (anhydro)	1013.4091	1013.4242	–14.9	17.8	+
GM-AEJF	1017.4433	1017.4347	8.4	–15.0	+
GM-AEJY	1033.4353	1033.4278	7.2	–5.3	+
GM-AEJAV	1040.4808	1040.4716	8.8	–14.7	+
GM-AEJIA	1054.4964	1054.4874	8.5	–11.3	+
GM-AEJW	1056.4394	1056.4455	–5.8	4.0	+
GM-AEJAM	1072.4524	1072.4460	5.9	–4.3	+
GM-AEJKR	1154.5667	1154.5631	3.1	–8.1	+
GM-GM-AE	1176.4836	1176.4590	20.9	–24.7	+
GM-GM-AEJ	1348.5684	1348.5457	16.9	–24.9	+
GM-GM-AEJA	1419.6055	1419.5824	16.2	–23.5	+
GM-AEJA-GM-AEJ (amidase product)	1313.5721	1313.5674	3.5	–11.0	+
GM-AEJA-GM-AEJA (amidase product)	1384.6092	1384.6037	4.0	–7.4	+
GM-AEJ-GM-AEJ (anhydro)	1702.7042	1702.6976	3.9	38.3	+
GM-AEJ-GM-AEJ	1722.7304	1722.7234	4.1	–8.6	+
GM-AEJA-GM-AEJ (double anhydro)	1753.7151	1753.7043	6.2	–7.2	+
GM-AEJA-GM-AEJ (anhydro)	1773.7413	1773.7339	4.2	–11.1	+
GM-AEJA-GM-AEJ	1793.7675	1793.7596	4.4	–8.8	+
GM-AEJA-GM-AEJA (dacetyl)	1822.7941	1822.7808	7.3	–7.4	+
GM-AEJA-GM-AEJA (double anhydro)	1824.7601	1824.7447	8.4	–15.6	+
GM-AEJA-GM-AEJA (anhydro)	1844.7784	1844.7704	4.3	–8.3	+
GM-AEJA-GM-AEJG	1850.7889	1850.8158	–14.6	9.7	+
GM-AEJA-GM-AEJA	1864.8046	1864.7962	4.5	–6.6	+
GM-AEJA-GM-AEJK (anhydro)	1901.8410	1901.8297	5.9	–14.5	+
GM-AEJA-GM-AEJL	1906.8562	1906.8452	5.8	–11.3	+
GM-AEJA-GM-AEJK	1921.8672	1921.8586	4.5	–12.0	+
GM-AEJA-GM-AEJF	1940.8402	1940.8263	7.2	–8.8	+
GM-AEJA-GM-AEJY	1956.8322	1956.8210	5.7	–7.6	+
GM-AEJA-GM-AEJAL	1977.8933	1977.8813	6.0	–10.7	+
GM-AEJA-GM-AEJKR	2077.9636	2077.9589	2.2	–13.0	+
GM-GM-AEJ-GM-AEJ	2200.9282	2200.9000	12.8	–17.7	+
GM-GM-AEJA-GM-AEJ	2271.9653	2271.9368	12.6	–18.4	+
GM-GM-AEJA-GM-AEJA	2343.0024	2342.9734	12.4	411.4	+
GM-AEJA-GM-AEJA-GM-AEJ (double anhydro)	2677.1120	2677.1000	4.5	–10.7	+
GM-AEJA-GM-AEJA-GM-AEJ (anhydro)	2697.1382	2697.1259	4.6	–8.6	+
GM-AEJA-GM-AEJA-GM-AEJ	2717.1644	2717.1532	4.1	–10.7	+
GM-AEJA-GM-AEJA-GM-AEJA (double anhydro)	2748.1491	2748.1363	4.7	–11.0	+
GM-AEJA-GM-AEJA-GM-AEJA (anhydro)	2768.1753	2768.1674	2.9	–11.2	+
GM-AEJA-GM-AEJA-GM-AEJA	2788.2015	2788.1919	3.4	–9.7	+
GM-AEJA-GM-AEJA-GM-AEJK (anhydro)	2825.2379	2825.2205	6.1	–9.3	+
GM-GM-AEJA-GM-AEJA-GM-AEJ	3195.3622	3195.3264	11.2	–14.0	+
GM-GM-AEJA-GM-AEJA-GM-AEJA	3266.3993	3266.3630	11.1	–12.5	+

Alternative structures were matched:.

GM-AEJ-GM-AEJK.

GM-AEJ-GM-AEJKA (anhydro).

GM-AEJ-GM-AEJKA.

GM-AEJ-GM-AEJA-GM-AEJKA (anhydro).

### A PG analysis workflow using freely available tools

The automated identification of *P. aeruginosa* muropeptides using PGFinder and the strategy reported previously (Anderson et al., 2020a; Anderson et al., 2020b) relied on commercially available deconvolution software (ProteinMetrics Byos or Agilent MassHunter, respectively). We sought to identify a free alternative software for mass deconvolution to make our PG analysis pipeline accessible to everyone. MaxQuant was selected as a tool of choice since it represents a widely used software package to analyse high-resolution mass-spectrometric data for shotgun proteomics (Cox and Mann, 2008). MaxQuant has native support for Thermo MS data (RAW) and Sciex (WIFF) file formats and supports the open MS data format mzXML. Virtually any proprietary MS data file can be converted to the mzXML open format using freely available tools such as Proteowizard and TOPPAS, making this workflow universally applicable. As proof of concept, we converted *P. aeruginosa* data to an mzXML file (Appendix 2—figure 1), processed it using MaxQuant for mass deconvolution (Appendix 2—figure 2), and analysed it using PGFinder for PG structure and composition identification. We were able to identify all the expected muropeptides (Table 2). This result confirms that the automated analysis of PG datasets can be carried out using the MaxQuant freeware and our open-source script PGFinder (Table 2 and Table 2—source data 2).

## Discussion

This study describes a workflow for the unbiased and automated analysis of bacterial PG using freely available resources. We analysed high-resolution MS datasets corresponding to PG fragments and demonstrated that this approach is a powerful tool to identify muropeptides and carry out comparative analyses based on the MS1 data.

MS analysis of bacterial PG has been carried out since the late 1980s (Garcia-Bustos et al., 1988; Martin et al., 1987). Whilst rp-HPLC-MS is now routinely used to explore PG structure, data analysis remains a manual process. Therefore, this step has become a bottleneck that prevents high-throughput analyses and introduces a series of issues regarding reproducibility. A major issue deals with the definition of the search space used since the list of muropeptides searched is not provided. Our previous work showed that an unbiased approach using shotgun proteomics tools identifies muropeptides containing unusual amino acids in *C. difficile* (Bern et al., 2017). Our unbiased search identified >106 masses matching *E. coli* muropeptide structures, representing a number strikingly larger than previously reported (Kühner et al., 2014). This level of complexity was anticipated based on the complement of enzymes involved in *E. coli* PG synthesis but had never been reported before. Our work therefore highlighted the limitations of search strategies reported so far, especially if we consider the fact that some bacterial PGs like *E. coli* have been extensively studied over the past 30 years.

Several issues and flaws associated with the manual analysis of PG MS data are addressed by our approach, which represents a robust, consistent, and open-access strategy. The PGFinder algorithm has been designed to create dynamic databases that are ultimately combined to perform the final matching process. Optimisation of the search space relies on a preliminary identification of masses matching theoretical monoisotopic masses of monomers, limiting misidentifications based on mass coincidence. Another advantage is that the only information provided by the user is a restricted monomer database rather than a comprehensive one. This avoids a time-consuming operation, prone to human error. PGFinder uses XIC for quantification of PG fragments, providing high resolution, sensitivity, and reproducibility, as indicated by the comparisons across biological replicates (Appendix 1—figure 2 and Figure 4a). It enables the accurate quantification of molecules with overlapping retention times and those present in very low abundance, with a large dynamic range (typically six orders of magnitude). Although a Matlab-based software package (Chromanalysis) has been described to automate the detection and quantification of UV peaks through Gaussian fitting (Desmarais et al., 2015), quantification using XIC is more straightforward. It is worth pointing out that the approach described here does not give absolute quantification of muropeptides. Instead, it allows a relative quantification of muropeptides. Nevertheless, this strategy remains suitable for comparative analyses and overcomes two major limitations associated with UV detection, namely detection threshold and co-elution of molecules.

Our *E. coli* PG analysis confirmed that PGFinder is a powerful tool that provides a much improved qualitative and quantitative PG analysis (Kühner et al., 2014; Morè et al., 2019). Combining an unbiased search with highly sensitive detection of individual structures is important for two reasons. Firstly, it opens the possibility to identify subtle modifications of PG structure, resulting from either a transient or a localised enzymatic activity such as that taking place at the septum. Secondly, it will permit the identification of previously undetected modifications that may provide new insights into our understanding of PG composition and dynamics. For example, we showed that *E. coli* PG contains a low abundance of deacetylated sugars. This observation is puzzling because no canonical PG deacetylase genes have been identified in this organism. Although the biological relevance of this property remains to be established, we cannot exclude the possibility that PG deacetylation in *E. coli* may contribute to PG homeostasis. Another striking outcome resulting from our automated search is the identification of a slightly higher amount of muropeptides containing anhydromuramic acid (4.55%) as compared to 2–3% (Glauner et al., 1988; Liu et al., 2020). To explain the discrepancy between our work and data from the literature, it is tempting to assume that most of the muropeptides containing anhydromuramic acid identified with PGFinder were simply not searched in previous studies. It is worth pointing out that none of the papers describing PG analysis published to date has reported the list of structures searched in the MS data analysed.

One of our objectives was to create an automated PG analysis tool accessible to the broadest audience possible, including people with no prior experience with programming or coding languages. Therefore, we shared PGFinder as a Jupyter Notebook allowing users to customise the search strategy depending on both the question asked and the instrument accuracy. PGFinder is particularly suitable for the characterisation of novel PGs with unknown composition or structural modifications and can be modified by users to add novel functionalities. However, a current limitation of this workflow is that it does not process MS/MS data. Therefore, the fragmentation spectra of individual monomers must be checked using dedicated tools to validate that the inferred structures are correct. We are currently working towards an integrated pipeline that includes MS/MS analysis to our PGFinder pipeline. The ability to disable some PG modifications means that the complexity of the search can be adjusted to focus on specific properties (e.g., the occurrence of acetylation/deacetylation, or amidation) or specific muropeptides resulting from lytic activities (e.g., unsubstituted MurNAc residues resulting from amidase activity). For PG that have already been well characterised (*E. coli*, *P. aeruginosa,* or *C. difficile*), the search parameters are already established, allowing a very straightforward analysis to be performed. Therefore, access to a custom, semi-quantitative sensitive analysis is ideal for comparing PG dynamics or differences in PG structure between a reference strain and isogenic mutants. Both reduced disaccharide peptides or lactyl-peptides (generated by beta-elimination) are identified using PGFinder.

We anticipate that an open access to PGFinder, in conjunction with freely available deconvolution tools, will allow researchers to carry out comparative MS1 analyses. The pipeline defined in this work enables reproducible and consistent data analysis. This represents the first step towards a standardised approach to PG analysis, opening the possibility to reanalyse datasets in repositories. The modular structure of the open-source PGFinder code can be easily integrated into any specific workflow for the automated processing of PG MS data.

## Materials and methods

**Key resources table:** 

Reagent type (species) or resource	Designation	Source or reference	Identifiers	Additional information
Strain, strain background(*Escherichia coli*)	BW25113	https://doi.org/10.1073/pnas.120163297	RRID:Addgene_72340	Model strain for PG analysis
Strain, strain background(*Clostridioides difficile*)	R20291	https://doi.org/10.1128/JB.0073107		Model strain for PG analysis
Strain, strain background(*Clostridioides difficile*)	M7404	https://doi.org/10.1371/journal.ppat.1002317		Model strain for PG analysis
Software, algorithm	PGFinderv.0.02	This work		Used for MS1 analysis of PG structure
Software, algorithm	Byosv.3.9–32	Protein Metrics Inc		Used for MS data deconvolution and MS/MS analysis
Software, algorithm	MaxQuant v2.0.1.0	Cox and Mann, 2008	RRID:SCR_014485	Used for MS data deconvolution
Software, algorithm	Perseusv.1.6.10.53	Tyanova et al., 2016	RRID:SCR_015753	Used statistical analysis of muropeptide abundance

### Bacterial strains and culture conditions

*E. coli* BW25113 was grown at 37°C in LB under agitation (250 rpm). *C. difficile* strains were cultured in heart infusion supplemented with yeast extract, L-cysteine, and glucose in an atmosphere of 10% H_2_, 10% CO_2_, and 80% N_2_ at 37°C in a Coy chamber or Don Whitley A300 anaerobic workstation.

### PG purification

PG was purified from exponential (*E. coli*) or late exponential (*C. difficile*) phase as described previously (Eckert et al., 2006; Glauner, 1988), freeze-dried, and resuspended in distilled water at a concentration of 5 mg/ml.

### Preparation of soluble muropeptides

PG (1 mg) was digested overnight with 25 µg of mutanolysin at 37°C in 150 µl of 20 mM sodium phosphate buffer (pH 5.5). Soluble disaccharide peptides were recovered in the supernatant following centrifugation (20,000 × *g* for 20 min at 25°C). To reduce muropeptides, equal volumes (200 µl) of the solution of disaccharide peptides and of borate buffer (250 mM, pH 9.0) were mixed. 2 ml of sodium borohydride was added, and the solution was incubated for 20 min at room temperature. The pH of the solution was adjusted to 4.0 with 20% orthophosphoric acid. Beta-elimination was carried out by mixing 200 µl of muropeptides with 64 µl of 32% (w/v) ammonia. After 5 hr at 37 C, the solution was neutralised with 60 µl of acetic acid glacial, freeze-dried, and resuspended in water (Arbeloa et al., 2004; Eckert et al., 2006).

The reduced muropeptides were desalted by reverse-phase high-performance liquid chromatography (rp-HPLC) on a C18 Hypersil Gold aQ column (3 µm, 2.1 × 200 mm; Thermo Fisher) at a flow rate of 0.4 ml/min. After 1 min in water-0.1% formic acid (v/v) (buffer A), muropeptides were eluted with a 6 min linear gradient to 95% acetonitrile-0.1% formic acid (v/v). Muropeptides were freeze-dried and resuspended in 100 µl. An aliquot of the desalted samples was analysed by rp-HPLC on the same column to measure the UV absorbance of the most abundant monomer (no isocratic step, muropeptides were eluted with a 30 min linear gradient to 15% acetonitrile-0.1% formic acid [v/v]). Samples were diluted to contain 150 mAU/µl of the major monomer and 10 µl were injected. Based on the dry weight of the PG sample, we estimated that this corresponded to approximately 50 µg of material.

### UHPLC-MS/MS

An Ultimate 3000 Ultra High-Performance Chromatography (UHPLC; Dionex/Thermo Fisher Scientific) system coupled with a high-resolution Q Exactive Focus mass spectrometer (Thermo Fisher Scientific) was used for LC/HRMS analysis. Muropeptides were separated using a C18 analytical column (Hypersil Gold aQ, 1.9 µm particles, 150 × 2.1 mm; Thermo Fisher Scientific), column temperature at 50°C. Muropeptides elution was performed by applying a mixture of solvent A (water, 0.1% [v/v] formic acid) and solvent B (acetonitrile, 0.1% [v/v] formic acid). After 10 µl sample injection, MS/MS data were acquired during a 40 min step gradient: 0–12.5% B for 25 min; 12.5–20% B for 5 min; held at 20% B for 5 min, and the column was re-equilibrated for 10 min under the initial conditions.

The Q Exactive Focus was operated under electrospray ionization (H-ESI II)-positive mode. Full scan (*m/z* 150–2250) used resolution 70,000 (FWHM) at *m/z* 200, with an automatic gain control (AGC) target of 1 × 10^6^ ions and an automated maximum ion injection time (IT).

Data-dependent MS/MS were acquired on a ‘Top 3’ data-dependent mode using the following parameters: resolution 17,500; AGC 1 × 10^5^ ions, maximum IT 50 ms, NCE 25%, and a dynamic exclusion time 5 s.

### MS data deconvolution

Byos search parameters to get .ftrs file Protein Metrics Byos v.3.9–32 was used to identify and compute the XICs. The parameters used for mass deconvolution using MaxQuant v.2.0.1.0 are described in Appendix 2—figure 2.

#### Data analysis

The crosslinking index and glycan chain length were calculated as described previously (Glauner, 1988). Label-free relative quantitation of muropeptides from triplicate *C. difficile* clinical isolates (R20291 and M7404) was performed using Byos 3.11, and statistical analysis of the quantitative data was performed using Perseus v. 1.6.10.53 (Tyanova et al., 2016). Briefly, muropeptide intensities were log_2_ transformed and normalised by subtraction of the median value. A two-sample Student’s *t*-test was performed with a permutation-based FDR of 0.05 to determine statistically significant quantitative differences between the strains. Comparisons between R20291 and M7404 muropeptide distribution (mono-, di-, tri-, tetra-pentamer) was evaluated for statistical significance using GraphPad Prism (unpaired *t*-test).

### Runtime environment

Code is available at https://github.com/Mesnage-Org/PGFinder (Patel, 2021). https://github.com/Mesnage-Org/PGFinder/releases/tag/v0.02 will take you to the archived release used in this paper. We used Python 3 to write the MS1 package and demonstrate its functionality using demo scripts. PGFinder can be run through an interactive Jupyter Notebook hosted on mybinder for ease of use by those less familiar with Python code. A conda environment is provided to ensure reproducible execution. Regression testing has been implemented to ensure changes to code do not cause changes to important results. The GitHub contains an interactive version to run user’s analysis and an end-to-end demo using samples data provided with the script (Interactive PGFinder). The sample data is a MaxQuant deconvolution output from the *E. coli* MS data analysed in the paper. The current version of the script can handle both .txt (MaxQuant) or .ftrs (Byos) deconvoluted data and offers the possibility for the user to include several modifications in the search. The time window for the ‘clean up step’ (in-source decay and salt adducts) as well as ppm tolerance for matching can also be defined by the user; the default values corresponding to these parameters used in this work are 0.5 min and 10 ppm.

### Data availability

All *E. coli* and *C. difficile* MS datasets generated in this study are available through the GlycoPOST repository (GPST000168; Watanabe et al., 2021). *P. aeruginosa* MS datasets are accessible via Figshare (Anderson et al., 2020b).

## Figures and Tables

**Appendix 1—figure 1.**
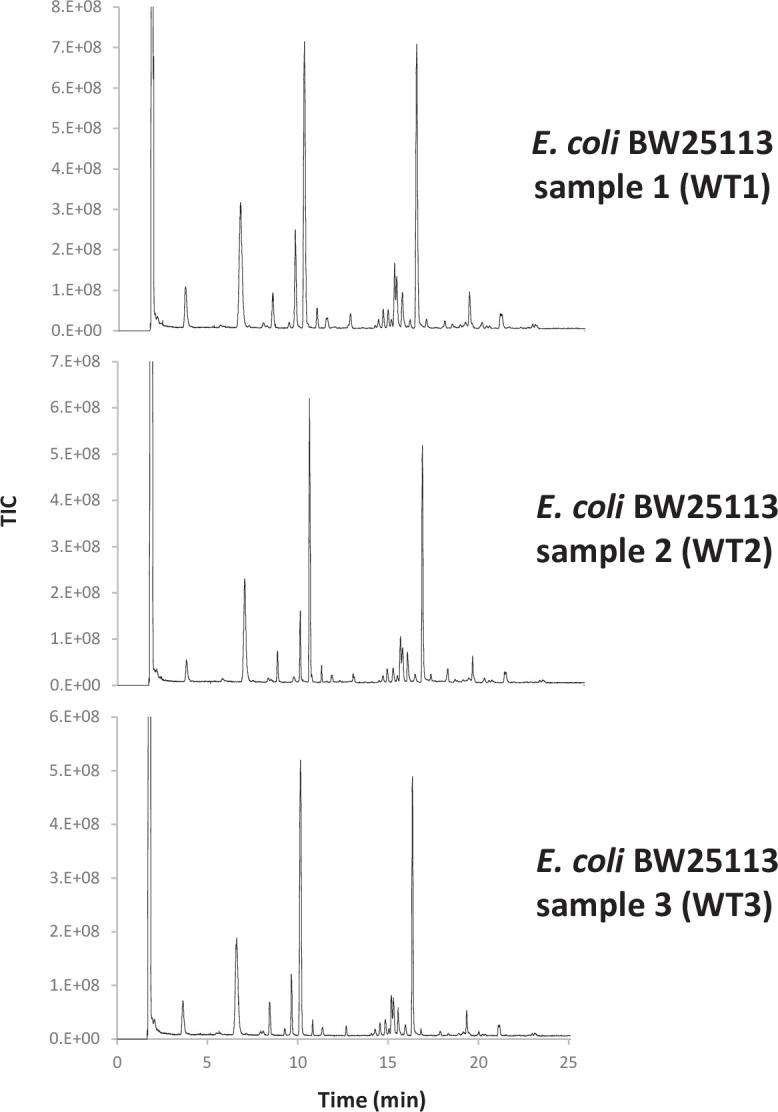
UHPLC-MS chromatogram of *E. coli* reduced disaccharide peptides.

**Appendix 1—figure 2.**
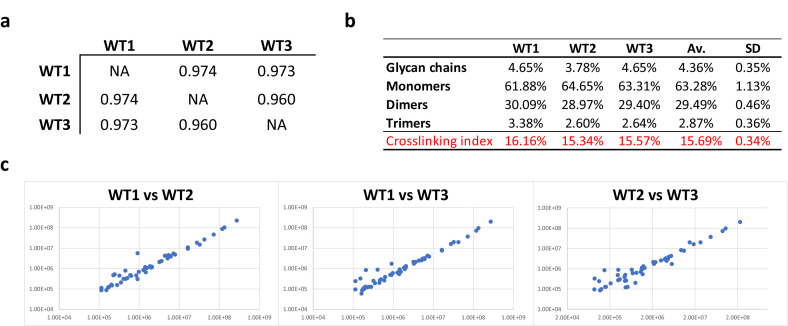
Consistency of *E. coli* PG analyses. (**a**) Pearson’s correlation coefficients across biological replicates of *E. coli* BW25113. (**b**) Muropeptide distribution according to degree of crosslinking. The crosslinking index was calculated as described previously (Glauner, 1988). (**c**) Pairwise comparisons of intensities corresponding to individual muropeptides identified in biological replicates. WT1, WT2 and WT3 correspond to individual biological replicates; Av., average abundance; SD, standard deviation.

**Appendix 2—figure 1.**
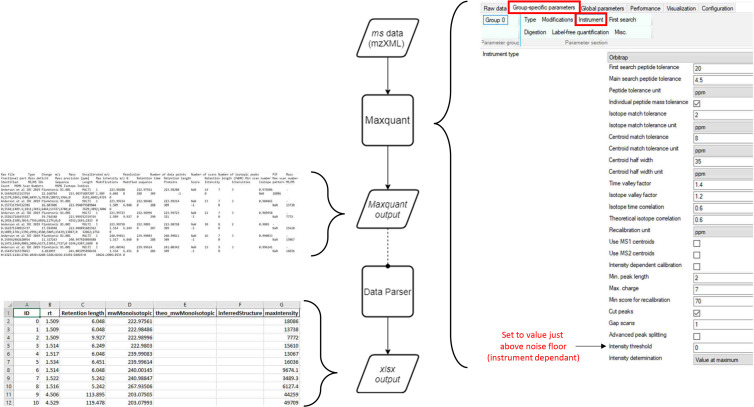
Workflow for production of MaxQuant compatible MS data files from Agilent QTOF data. Agilent MS data (data: .d) is converted by Proteowizard to a mzML format (data: XML). Relevant settings for Proteowizard are shown (left). mzML file is then converted by TOPPAS to a mzXML file (data: XML). Relevant settings are shown (right).

**Appendix 2—figure 2.**
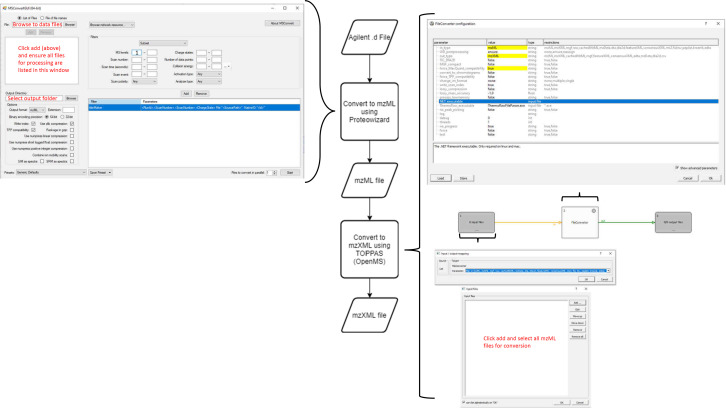
Workflow for MS data processing using MaxQuant, before automated analysis. mzXML (data: XML) is passed to MaxQuant (process) for deconvolution and monoisotopic mass determination. Default values used except where indicated (right). MaxQuant output (data: text file) is then passed to the data parser module (process). This module removes superfluous data and reformats remaining data to be compatible with the matching script as an Excel file (data: xlsx).

## Data Availability

All raw mass spectrometry data files have are available through the Glycopost repository (ref GPST000168). The following previously published datasets were used: AndersonEM SychanthaD BrewerD ClarkeAJ Geddes-McAlisterJ KhursigaraCM 2020Peptidoglycomics: Examining compositional changes in peptidoglycan between biofilm- and planktonic-derived *Pseudomonas aeruginosa*figshare10.6084/m9.figshare.10277909PMC695653131771981

## Additional files

Transparent reporting form

Supplementary file 1. Step by step strategy for PG analysis.

## References

[bib1] Anderson EM, Greenwood NA, Brewer D, Khursigara CM (2020a). Semi-quantitative analysis of peptidoglycan by liquid chromatography mass spectrometry and bioinformatics. Journal of Visualized Experiments.

[bib2] Anderson EM, Sychantha D, Brewer D, Clarke AJ, Geddes-McAlister J, Khursigara CM (2020b). Peptidoglycomics reveals compositional changes in peptidoglycan between biofilm- and planktonic-derived *Pseudomonas aeruginosa*. The Journal of Biological Chemistry.

[bib3] Arbeloa A, Hugonnet JE, Sentilhes AC, Josseaume N, Dubost L, Monsempes C, Blanot D, Brouard JP, Arthur M (2004). Synthesis of mosaic peptidoglycan cross-bridges by hybrid peptidoglycan assembly pathways in gram-positive bacteria. J Biol Chem.

[bib4] Bern M, Beniston R, Mesnage S (2017). Towards an automated analysis of bacterial peptidoglycan structure. Anal Bioanal Chem.

[bib5] Boneca IG, Dussurget O, Cabanes D, Nahori M-A, Sousa S, Lecuit M, Psylinakis E, Bouriotis V, Hugot J-P, Giovannini M, Coyle A, Bertin J, Namane A, Rousselle J-C, Cayet N, Prévost M-C, Balloy V, Chignard M, Philpott DJ, Cossart P, Girardin SE (2007). A critical role for peptidoglycan N-deacetylation in *Listeria* evasion from the host innate immune system. PNAS.

[bib6] Cox J, Mann M (2008). MaxQuant enables high peptide identification rates, individualized p.p.b.-range mass accuracies and proteome-wide protein quantification. Nature Biotechnology.

[bib7] Cummins CS, Harris H (1956). The chemical composition of the cell wall in some gram-positive bacteria and its possible value as a taxonomic character. Journal of General Microbiology.

[bib8] Desmarais SM, Tropini C, Miguel A, Cava F, Monds RD, de Pedro MA, Huang KC (2015). High-throughput, highly sensitive analyses of bacterial morphogenesis using ultra performance liquid chromatography. The Journal of Biological Chemistry.

[bib9] Eckert C, Lecerf M, Dubost L, Arthur M, Mesnage S (2006). Functional analysis of AtlA, the major N-acetylglucosaminidase of *Enterococcus faecalis*. Journal of Bacteriology.

[bib10] Garcia-Bustos JF, Chait BT, Tomasz A (1988). Altered peptidoglycan structure in a pneumococcal transformant resistant to penicillin. Journal of Bacteriology.

[bib11] Glauner B (1988). Separation and quantification of muropeptides with high-performance liquid chromatography. Analytical Biochemistry.

[bib12] Glauner B, Höltje JV, Schwarz U (1988). The composition of the murein of *Escherichia coli*. The Journal of Biological Chemistry.

[bib13] Harz H, Burgdorf K, Höltje JV (1990). Isolation and separation of the glycan strands from murein of *Escherichia coli* by reversed-phase high-performance liquid chromatography. Analytical Biochemistry.

[bib14] Juan C, Torrens G, Barceló IM, Oliver A (2018). Interplay between peptidoglycan biology and virulence in gram-negative pathogens. Microbiology and Molecular Biology Reviews.

[bib15] Kühner D, Stahl M, Demircioglu DD, Bertsche U (2014). From cells to muropeptide structures in 24 h: Peptidoglycan mapping by UPLC-MS. Scientific Reports.

[bib16] Liu X, Biboy J, Consoli E, Vollmer W, den Blaauwen T (2020). Mrec and MRED balance the interaction between the elongasome proteins pbp2 and RODA. PLOS Genetics.

[bib17] Mainardi JL, Villet R, Bugg TD, Mayer C, Arthur M (2008). Evolution of peptidoglycan biosynthesis under the selective pressure of antibiotics in gram-positive bacteria. FEMS Microbiology Reviews.

[bib18] Martin SA, Rosenthal RS, Biemann K (1987). Fast atom bombardment mass spectrometry and tandem mass spectrometry of biologically active peptidoglycan monomers from *Neisseria gonorrhoeae*. The Journal of Biological Chemistry.

[bib19] Morè N, Martorana AM, Biboy J, Otten C, Winkle M, Serrano CKG, Montón Silva A, Atkinson L, Yau H, Breukink E, den Blaauwen T, Vollmer W, Polissi A (2019). Peptidoglycan remodeling enables *Escherichia coli* to survive severe outer membrane assembly defect. MBio.

[bib20] Mudd S, Lackman DB (1941). Bacterial morphology as shown by the electron microscope: I. Structural differentiation within the streptococcal cell. Journal of Bacteriology.

[bib21] Patel AV (2021). GitHub.

[bib22] Rogers HJ, Perkins HR (1959). Cell-wall mucopeptides of *Staphyloccus aureus* and *Micrococcus lysodeikticus*. Nature.

[bib23] Schleifer KH, Kandler O (1972). Peptidoglycan types of bacterial cell walls and their taxonomic implications. Bacteriological Reviews.

[bib24] Tipper DJ (2002). Alkali-catalyzed elimination of D-lactic acid from muramic acid and its derivatives and the determination of muramic acid. Biochemistry.

[bib25] Turner RD, Mesnage S, Hobbs JK, Foster SJ (2018). Molecular imaging of glycan chains couples cell-wall polysaccharide architecture to bacterial cell morphology. Nature Communications.

[bib26] Tyanova S, Temu T, Sinitcyn P, Carlson A, Hein MY, Geiger T, Mann M, Cox J (2016). The Perseus computational platform for comprehensive analysis of (prote)omics data. Nature Methods.

[bib27] Vollmer W, Blanot D, de Pedro MA (2008). Peptidoglycan structure and architecture. FEMS Microbiology Reviews.

[bib28] Watanabe Y, Aoki-Kinoshita KF, Ishihama Y, Okuda S (2021). Glycopost realizes fair principles for glycomics mass spectrometry data. Nucleic Acids Research.

[bib29] Weidel W, Pelzer H (1964). Bagshaped macromolecules - a new outlook on bacterial cell walls. Advances in Enzymology and Related Subjects of Biochemistry.

[bib30] Wheeler R, Chevalier G, Eberl G, Gomperts Boneca I (2014). The biology of bacterial peptidoglycans and their impact on host immunity and physiology. Cellular Microbiology.

