# Supplementary material for: PGFinder, a novel analysis pipeline for the consistent, reproducible, and high-resolution structural analysis of bacterial peptidoglycans
Source: eLife. 2021 Sep 28;10:e70597. doi: 10.7554/eLife.70597 (PMC8478412; doi:10.7554/eLife.70597)
Supplement: Table 1—source data 3. [file elife-70597-table1-data3.pdf]

Table 1—source data 3. MS/MS analysis of *E. coli* glycan chains and monomers.

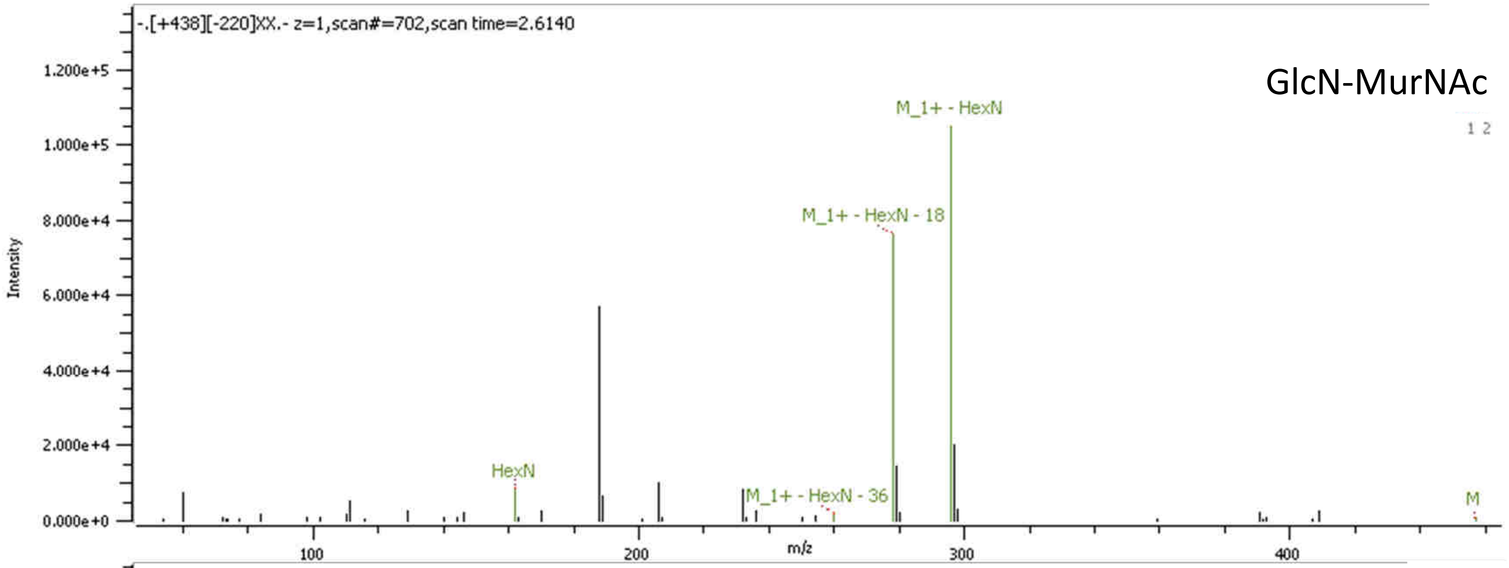

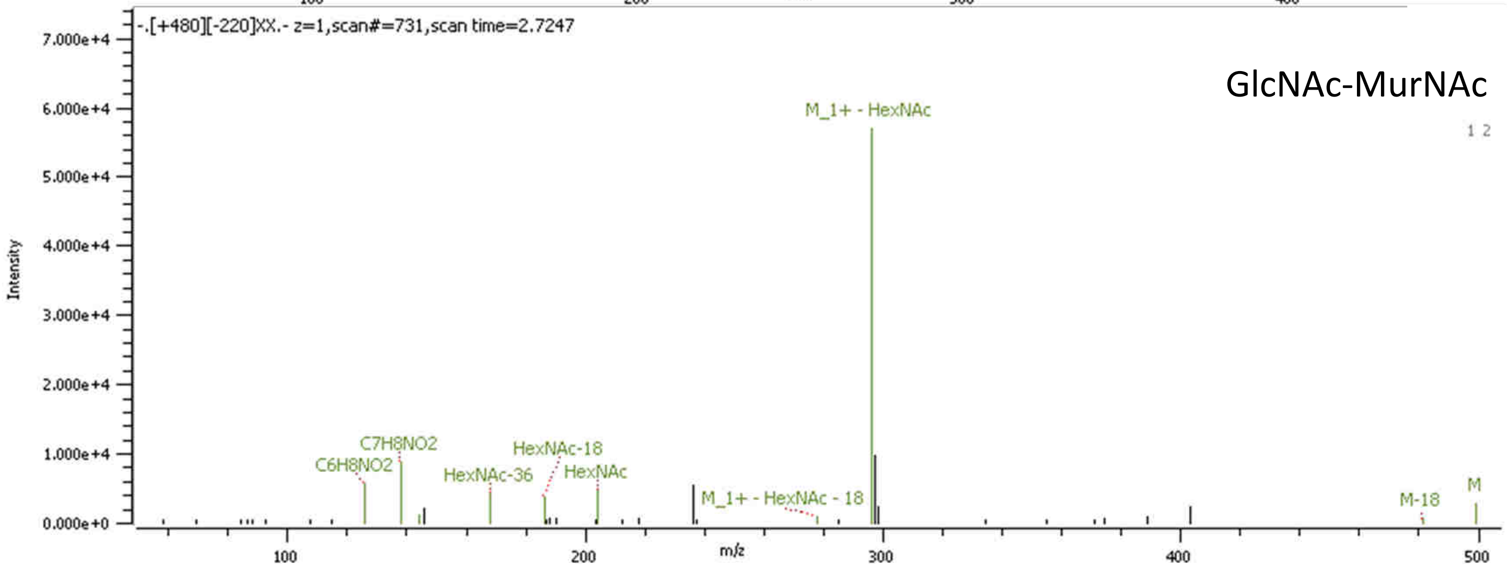

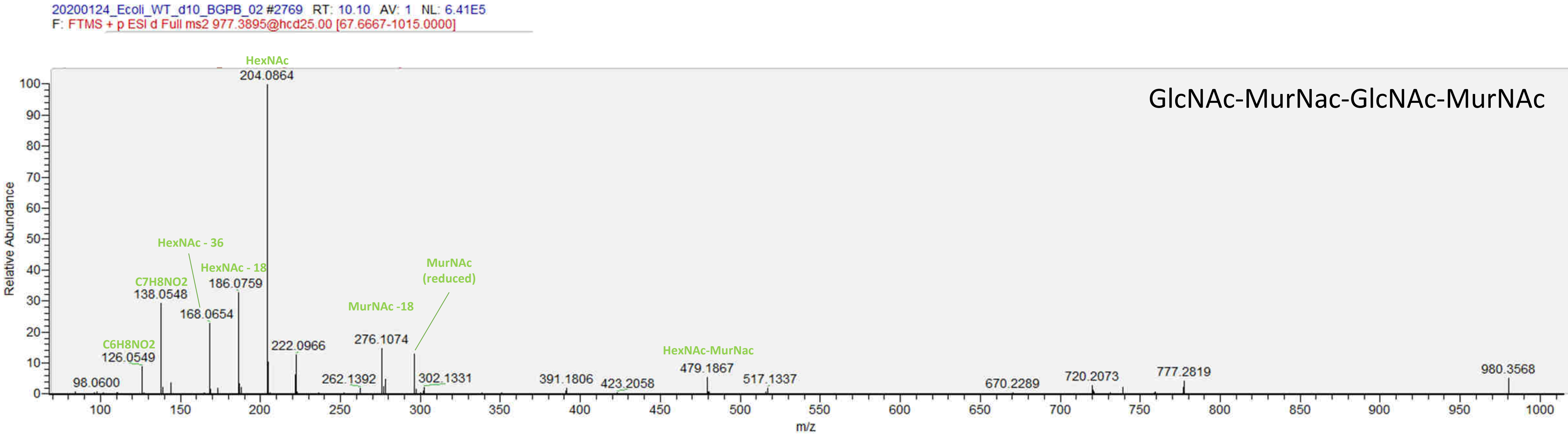
20200124_Ecoli_WT_d10_BGPB_02 #2769 RT: 10.10 AV: 1 NL: 6.41E5
F: FTMS + p ESI d Full ms2 977.3895@hcd25.00 [67.6667-1015.0000]
GlcNAc-MurNac-GlcNAc-MurNAc
HexNAc
204.0864
HexNAc - 36
HexNAc - 18
186.0759
C7H8NO2
138.0548
168.0654
C6H8NO2
126.0549
98.0600
222.0966
MurNAc -18
276.1074
MurNAc
(reduced)
262.1392
302.1331
391.1806
423.2058
HexNAc-MurNac
479.1867
517.1337
670.2289
720.2073
777.2819
980.3568
Relative Abundance
m/z

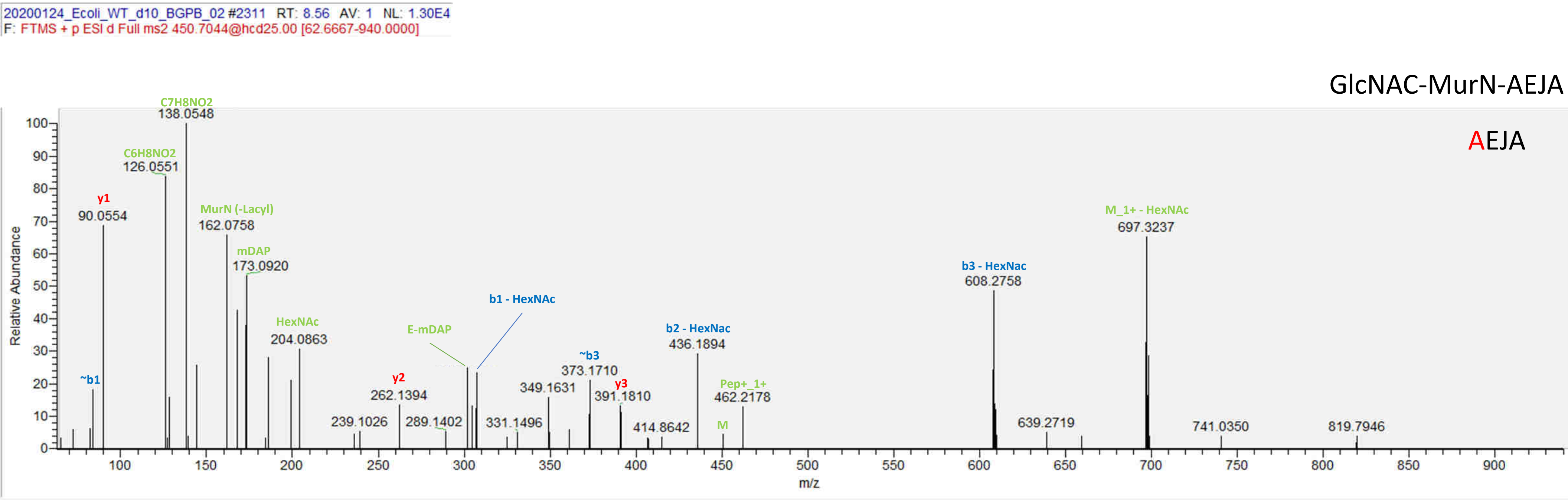

20200124_Ecoli_WT_d10_BGPB_02 #2311 RT: 8.56 AV: 1 NL: 1.30E4
F: FTMS + p ESI d Full ms2 450.7044@hcd25.00 [62.6667-940.0000]
GlcNAC-MurN-AEJA
AEJA
Relative Abundance
m/z
C7H8NO2
138.0548
C6H8NO2
126.0551
y1
90.0554
MurN (-Lacyl)
162.0758
mDAP
173.0920
HexNAc
204.0863
E-mDAP
b1 - HexNAc
~b1
y2
262.1394
239.1026
289.1402
331.1496
349.1631
~b3
373.1710
y3
391.1810
414.8642
b2 - HexNac
436.1894
Pep+_1+
462.2178
M
b3 - HexNac
608.2758
639.2719
M_1+ - HexNAc
697.3237
741.0350
819.7946

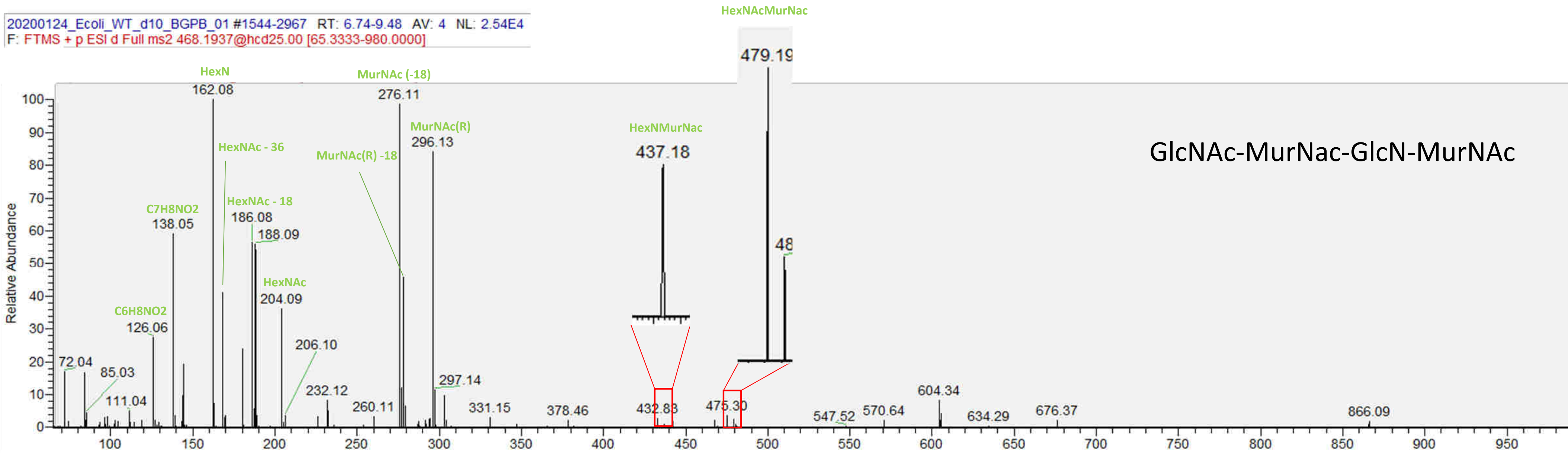

20200124_Ecoli_WT_d10_BGPB_01 #1544-2967 RT: 6.74-9.48 AV: 4 NL: 2.54E4
F: FTMS + p ESI d Full ms2 468.1937@hcd25.00 [65.3333-980.0000]
HexNAcMurNac
479.19
HexN
162.08
MurNAc (-18)
276.11
MurNAc(R)
296.13
HexNMurNac
437.18
GlcNAc-MurNac-GlcN-MurNAc
HexNAc - 36
MurNAc(R) -18
HexNAc - 18
186.08
C7H8NO2
138.05
188.09
48
HexNAc
204.09
C6H8NO2
126.06
206.10
72.04
85.03
111.04
232.12
260.11
297.14
331.15
378.46
432.83
475.30
547.52
570.64
604.34
634.29
676.37
866.09
Relative Abundance

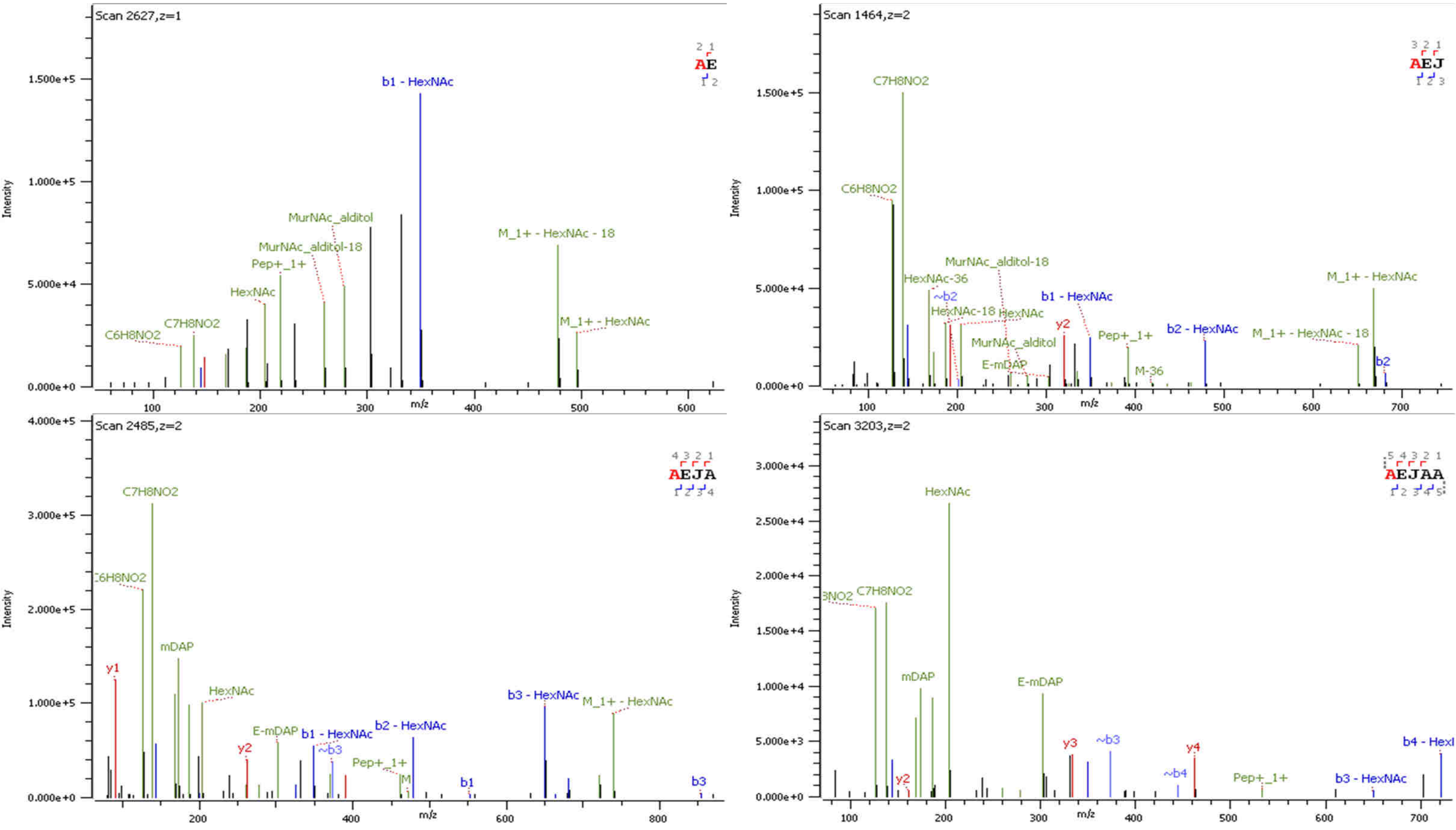

Scan 2627,z=1
AE
b1 - HexNAc
MurNAc_alditol
MurNAc_alditol-18
Pep+_1+
HexNAc
C7H8NO2
C6H8NO2
M_1+ - HexNAc - 18
M_1+ - HexNAc
Intensity
m/z
Scan 1464,z=2
AEJ
C7H8NO2
C6H8NO2
MurNAc_alditol-18
HexNAc-36
~b2
HexNAc-18
HexNAc
MurNAc_alditol
E-mDAP
b1 - HexNAc
y2
Pep+_1+
b2 - HexNAc
M-36
M_1+ - HexNAc
M_1+ - HexNAc - 18
b2
Scan 2485,z=2
AEJA
C7H8NO2
mDAP
y1
HexNAc
E-mDAP
y2
b1 - HexNAc
~b3
b2 - HexNAc
Pep+_1+
M
b1
b3 - HexNAc
M_1+ - HexNAc
b3
Scan 3203,z=2
AEJAA
HexNAc
C7H8NO2
mDAP
E-mDAP
y2
y3
~b3
~b4
y4
Pep+_1+
b3 - HexNAc

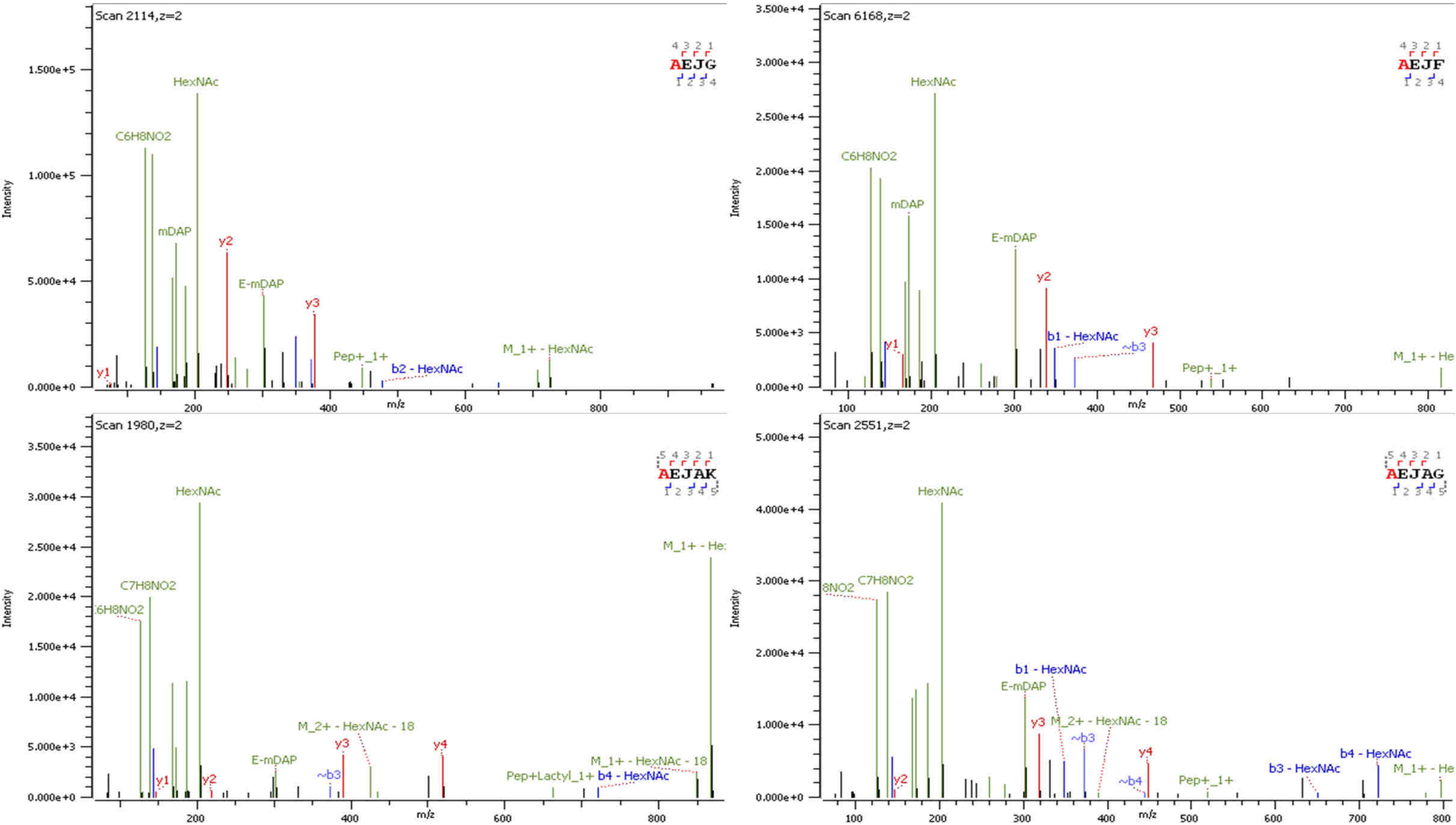

Scan 2114,z=2
AEJG
Scan 6168,z=2
AEJF
Scan 1980,z=2
AEJAK
Scan 2551,z=2
AEJAG
Intensity
m/z
HexNAc
C6H8NO2
C7H8NO2
mDAP
E-mDAP
Pep+_1+
Pep+Lactyl_1+
b1 - HexNAc
b2 - HexNAc
b3 - HexNAc
b4 - HexNAc
M_1+ - HexNAc
M_1+ - HexNAc - 18
M_2+ - HexNAc - 18

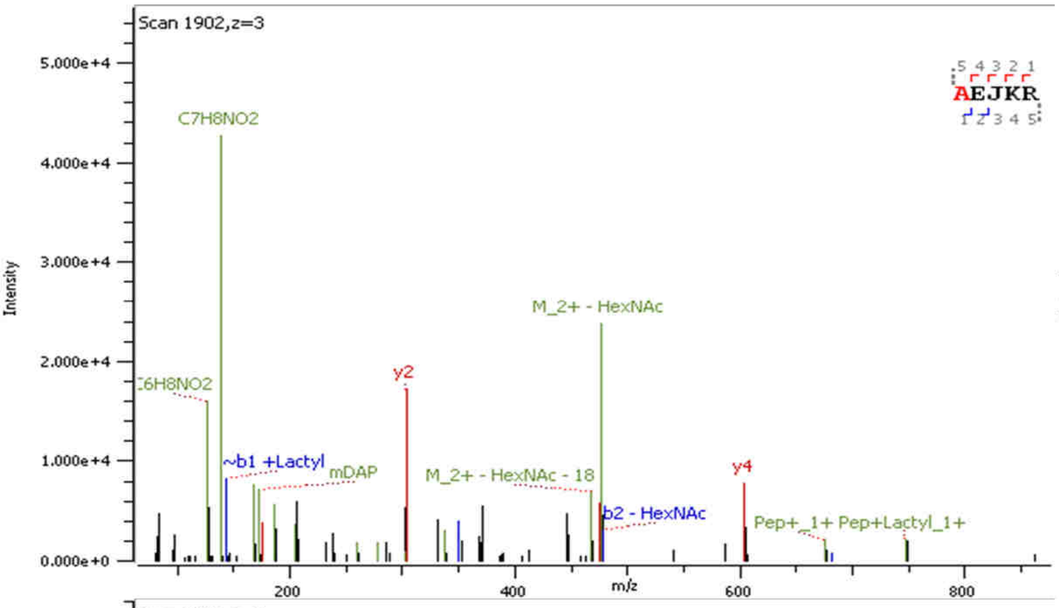
Scan 1902,z=3
AEJKR
Intensity
m/z
C7H8NO2
:6H8NO2
~b1 +Lactyl
mDAP
y2
M_2+ - HexNAc
M_2+ - HexNAc - 18
b2 - HexNAc
y4
Pep+_1+ Pep+Lactyl_1+

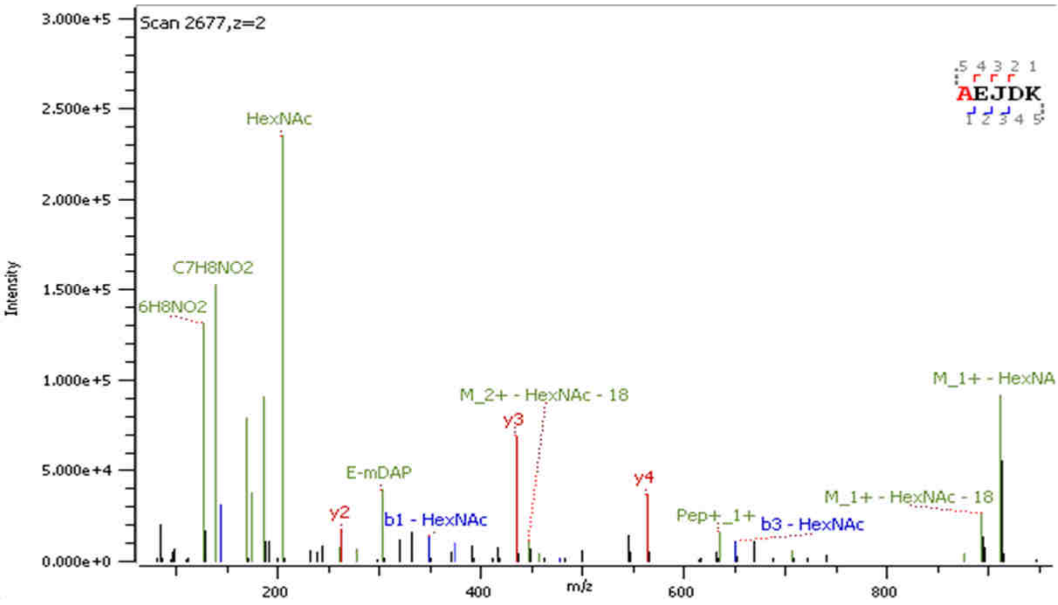
Scan 2677,z=2
AEJDK
Intensity
m/z
HexNAc
C7H8NO2
6H8NO2
E-mDAP
y2
b1 - HexNAc
y3
M_2+ - HexNAc - 18
y4
Pep+_1+
b3 - HexNAc
M_1+ - HexNAc - 18
M_1+ - HexNA

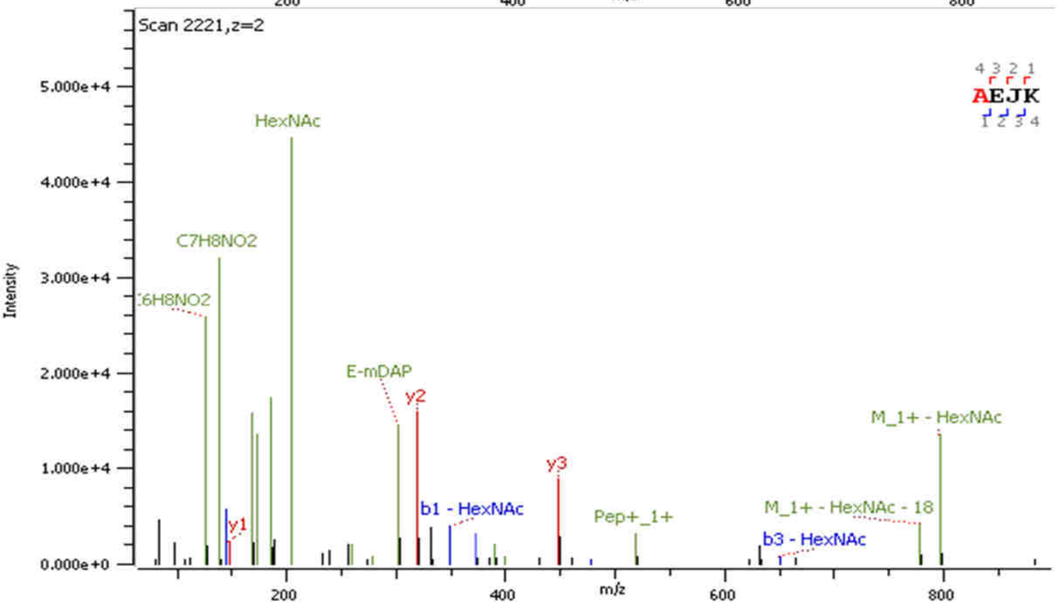
Scan 2221,z=2
AEJK
Intensity
m/z
HexNAc
C7H8NO2
:6H8NO2
E-mDAP
y2
y1
b1 - HexNAc
y3
Pep+_1+
M_1+ - HexNAc - 18
b3 - HexNAc
M_1+ - HexNAc
