# Supplementary material for: PGFinder, a novel analysis pipeline for the consistent, reproducible, and high-resolution structural analysis of bacterial peptidoglycans
Source: eLife. 2021 Sep 28;10:e70597. doi: 10.7554/eLife.70597 (PMC8478412; doi:10.7554/eLife.70597)
Supplement: Supplementary file 1. [file elife-70597-supp1.pdf]

1. Analyse datasets using PGFinder (1 csv file per MS data file)

2. Import each csv file into a single Excel workbook

Each imported csv file will correspond to an individual sheet with raw matched data

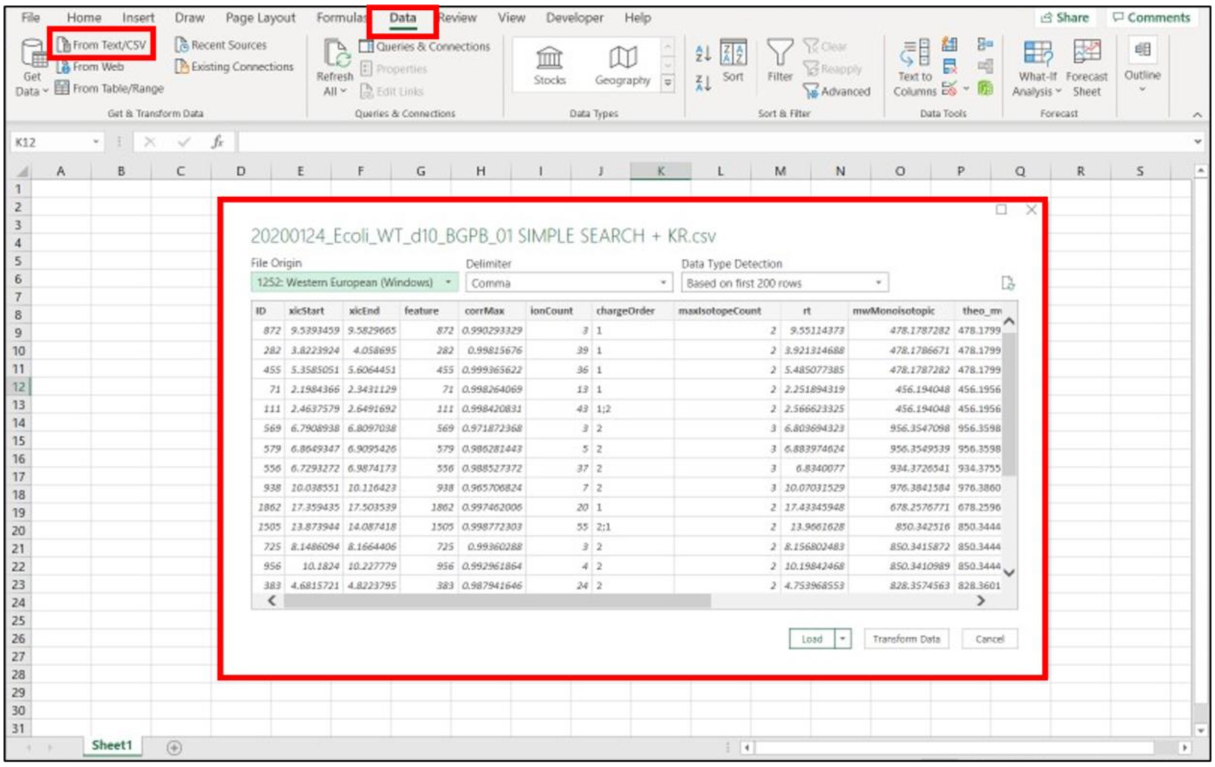

3. Consolidate (sum) the max intensity of muropeptides for each imported csv file

Merge repeated inferred structures into one entry, using inferred structure column as the basis for consolidation. This will sum the max intensity of the same structure across multiple retention times. The function must be set to sum.

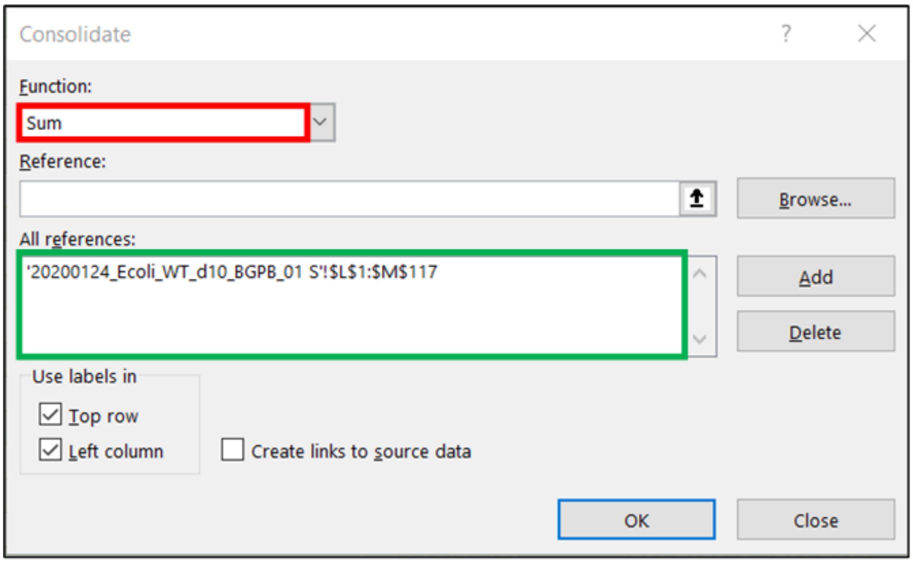

Referenced cells in green correspond to raw matched data (inferred structure and max intensity columns)

4. Remove all coincidental inferred structures

For masses matched with more than one inferred structure, use MS/MS data to determine the correct structure and remove all others.

5. Assign RT, theoretical and observed masses to inferred structures in consolidated data

Parameters above are assigned based on the inferred structure's most abundant recorded intensity in the raw matched data.

7. Consolidate (average & standard deviation) all biological replicate data

Merge averaged consolidated technical replicates for each sample into one entry, using inferred structure column as the basis for consolidation. The function must first set to average. This will return the average max intensity, rt, abundance, theoretical and observed mass across each averaged replicate. Repeat the consolidation described above but with the function set to standard deviation (population) to obtain the S.D for each parameter.

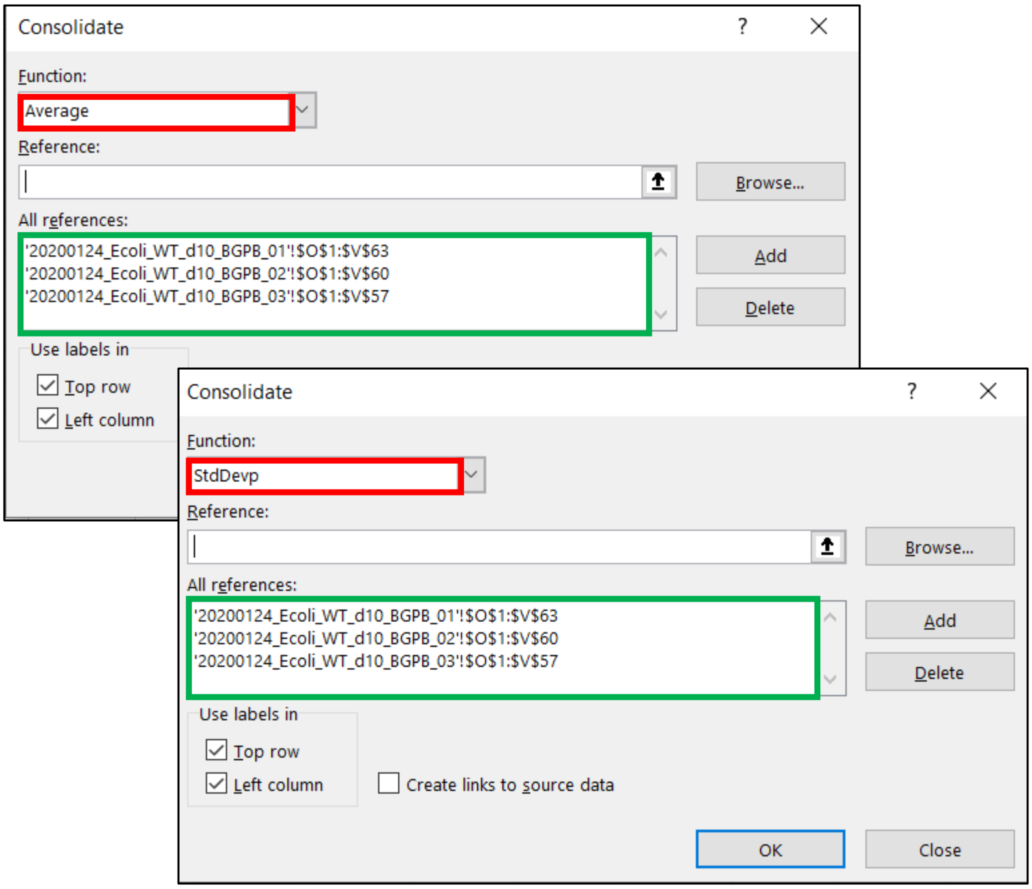

Referenced cells in green correspond to consolidated data for each replicate
